# Differences in Perceptions of Major Depressive Disorder Symptoms and Treatment Priorities Between Patients and Health Care Providers Across the Acute, Post-Acute, and Remission Phases of Depression

**DOI:** 10.3389/fpsyt.2019.00335

**Published:** 2019-05-21

**Authors:** Bernhard T. Baune, Michael Cronquist Christensen

**Affiliations:** ^1^Department of Psychiatry and Psychotherapy, University of Münster, Münster, Germany; ^2^Department of Psychiatry, Melbourne Medical School, University of Melbourne, Melbourne, VIC, Australia; ^3^Discipline of Psychiatry, Adelaide Medical School, The University of Adelaide, Adelaide, SA, Australia; ^4^H. Lundbeck A/S, Valby, Denmark

**Keywords:** major depressive disorder, psychosocial functioning, cognitive symptoms, phase of treatment, acute, post-acute, remission

## Abstract

Limited data exist on concordance between patients’ and health care providers’ (HCPs) perceptions regarding symptoms of major depressive disorder (MDD) and treatment priorities, particularly across disease phases. This study examined concordance during the acute, post-acute, and remission phases of MDD. In an online survey, 2,008 patients responded based on their experience with MDD, and 1,046 HCPs responded based on their clinical experience treating patients with MDD. Questions included symptom frequency and severity, treatment priorities, and impact on psychosocial functioning. Patients reported more frequently mood, physical, and cognitive symptoms than HCPs in the post-acute and remission phases and greater impact on psychosocial functioning. Patients reported that all these symptoms require high treatment priority across the phases of MDD, generally to a greater extent than HCPs. Patients also gave high emphasis to addressing impairment in psychosocial functioning early in the treatment course. A substantial difference in the effectiveness of treating symptoms of MDD between patients and HCPs was observed. This is the first study to quantify, broadly, differences in perceptions of MDD symptom prevalence, severity, and treatment priorities across MDD phases, and the study findings highlight a need for improved communication between patients and HCPs about symptoms, their impact on psychosocial functioning, and treatment priorities across phases.

## Introduction

Major depressive disorder (MDD) is a multifaceted condition (1) with emotional (e.g., feelings of worthlessness or diminished interest in life) (2), cognitive (e.g., trouble concentrating) (2, 3), and physical symptoms (e.g., insomnia or fatigue) (2), which have been shown to be important for psychosocial functioning. The functional impairment observed in patients with MDD extends to work, social, and family life and has important consequences for their health-related quality of life (4, 5).

Health care providers’ (HCPs) perceptions of treatment goals have traditionally focused on achieving full remission (6, 7) or on ameliorating mood symptoms (2), with the objective of removing depressive symptoms and reducing the risk of subsequent depressive episodes (6). Patients, on the other hand, appear to also focus on their psychosocial functioning, such as improving social and family relationships, increasing physical health behaviors, securing a job, and organizing the home (8).

There are limited data on the concordance and differences between patients’ and HCPs’ perceptions of the symptoms of MDD, their severity, and priority of treatment outcomes (6, 9). A study of patients’ perceptions of depressive symptoms, anxiety, psychosocial functioning, and health-related quality of life revealed that approximately half of the patients did not consider themselves to be in remission, despite meeting the symptom-based definition of remission according to the Hamilton Depression Rating Scale (10). Importantly, these patients also reported higher levels of depression and anxiety, greater functional impairment, and worse quality of life than the MDD patients who considered themselves in remission. Another more recent study by Marando et al. (11) in patients with MDD and comorbid human immunodeficiency virus reported similar remarkable differences between patients’ observations of depressive symptoms and physician assessment: using the self-reported Center for Epidemiological Studies-Depression (CES-D) 20-item scale, approximately 22% of the patients reported symptoms equaling severe depression, whereas only 4% were identified as having depression by their physician (11). The differences observed are important in clinical practice for both the diagnosis and management of MDD. A lower rating of depressive symptoms by physicians may lead them to declare remission too soon, while, despite some improvement, patients are still experiencing clinically relevant symptoms and functional impairment.

In addition to the mood and physical symptoms of MDD, a growing body of evidence has pointed to a high prevalence of cognitive symptoms experienced by patients with MDD, both in the acute and remission phases, as well as the need for identifying and managing these symptoms (3, 12, 13). Cognitive symptoms appear to play a critical role for patient functioning, including work functioning, and their presence in patients considered to be in remission has been associated with an increased risk of a relapse (14–16). So far, little is known about the perceived importance of cognitive symptoms and the implications for psychosocial functioning from a patient and HCP perspective. A clearer understanding of the perceived symptoms of MDD in the different phases of the disease, their impact on patient functioning, and resulting treatment priorities from the MDD patient and the treating HCP perspectives may improve patient–physician communication and ultimately clinical decision making.

The objectives of this research were to identify differences and similarities in perceptions of MDD symptoms, their impact on psychosocial functioning, and treatment goals among patients and HCPs across the different phases of MDD (acute, post-acute, and remission). Specifically, this study investigated the perspective of patients with MDD and treating HCPs in each of the three phases of MDD on: 1) presence and severity of depressive symptoms, as well as which symptoms they most want treated in each phase of MDD; 2) impact of MDD on patients’ ability to function at work, at home, and socially; and 3) treatment priorities, perceptions of treatment adequacy, and communication of treatment goals.

## Methods

### Study Design

This survey was conducted in Brazil, Canada, Mexico, South Korea, United States, France, Italy, and Spain between February 14, 2017, and March 28, 2017, in patients with MDD and HCPs regarding their perceptions of symptom prevalence, severity, impact on psychosocial functioning, and treatment goals. Respondents were recruited using online panels of consumers and HCPs where all panelists had provided consent to participate in the research. Their consent was sought again specifically for this survey. An online questionnaire was developed to allow for both screening of respondents and collection of the quantitative data needed to answer the specific research questions. Collection of online survey data is governed by strict data protection laws ensuring the anonymity of the respondents. Invitations to participate in the survey were sent to a total of 14,048 individuals identified as having depression in an initial screening (550 in Brazil, 605 in Canada, 1,375 in Mexico, 3,843 in South Korea, 492 in the United States, 537 in France, 3,923 in Italy, and 2,723 in Spain), of whom 2,379 patients took part in the survey (16.9%). For HCPs, a total of 2,428 psychiatrists, primary care physicians (PCPs), and, in Italy, neurologists, were invited to participate in the survey: 376 in Brazil, 431 in Canada, 282 in Mexico, 207 in South Korea, 363 in the United States, 281 in France, 290 in Italy, and 198 in Spain. Of these, 1,223 (50.4%) HCPs took part in the survey.

Patients were recruited for this survey based on their existing profile indicating a history of depression in the past 12 months (when information was already available). In addition, patients affirmed that they 1) had ever been diagnosed with depression by a physician, 2) were currently using prescribed medication for their depression, and 3) had used medication to treat depression in the past 3 months. For HCPs to take part in the survey, each HCP had to undergo a rigorous authentication process that involved validating personal details *via* a photocopied attachment of ID, email address, and medical license number (specific to the country of practice) and partaking in an introductory phone call. Of note, patients who completed the patient survey were not the same patients whom HCPs referred to during the HCP survey. Data protection law prevents matching samples of patients and HCPs. All participants were offered reward points for their participation in the survey. Reward points could be exchanged or redeemed for PayPal, vouchers (e.g., Amazon and iTunes), air miles, and donation to charity (e.g., UNICEF).

Before study initiation, a small pilot study was conducted with five HCPs and five patients in the United Kingdom to evaluate the questionnaires administered. The pilot study specifically examined the following: whether the questions were relevant and made sense, if the terminology was appropriate (e.g., definitions for acute, post-acute, and remission; see **Supplementary Appendix A Table 1**), and if the administration time was feasible; evaluated the ease of navigation in the questionnaire; and ascertained suggestions for improvement. All respondents reacted favorably to the questionnaire, and all physicians agreed with the definitions for each of the three phases of depression. The questionnaires used in each country were translated into the local language by native speakers and further assessed by a second native speaker. Translated questionnaires were then checked a final time before being approved for use in each country.

### Inclusion Criteria

Patients and HCPs were screened for eligibility before participating in the survey. Specific screening questions asked patients if they had ever been diagnosed with depression, were currently using a medication to treat their depression, or, if not currently taking medication, had used a medication to treat depression in the past 3 months. For inclusion, patients were required to carefully review and indicate which one of the following descriptions best described the phase of depression they were currently experiencing: 1) acute phase: “A time when your symptoms are at their worst or most severe and for which you use antidepressant treatment”; 2) post-acute phase: “A time when your symptoms are starting to improve, are less severe than they were before and you still use antidepressant treatment”; or 3) remission phase: “A time when your symptoms have improved significantly and you are already feeling better, but you may or may not still experience some minor symptoms. You may or may not be using antidepressant treatment.” Many questions throughout the interview referenced and provided the definition of whichever phase the patient had indicated at inclusion.

Three types of HCPs were recruited into this study: PCPs, psychiatrists, and neurologists. All HCPs were required to have treated and managed a minimum number of MDD patients per month: ≥15 for PCPs (in Brazil ≥10 patients), ≥40 for psychiatrists, and ≥25 for neurologists. HCPs were also required to spend ≥75% of their working hours in direct patient care and to be prescribing antidepressants to ≥75% of their MDD patients. In the United States, ≥90% of the HCPs’ patients had to be living outside of long-term care facilities. The aim was to obtain a good representation of all three types of HCPs in the sample; however, an exact ratio between these groups was not determined *a priori*.

All HCPs were also provided with clinically oriented definitions of each of the three phases of depression, and for inclusion, they were asked whether or not they could refer to three individual patient case records during the interview that matched these definitions. Specifically, each patient case record would represent a different patient type according to the phase of depression: 1) a patient in the acute phase of the condition: “By acute phase, we mean a patient experiencing acute symptoms of MDD that require antidepressant treatment”; 2) a patient in the post-acute phase of the condition: “By post-acute phase, we mean a phase where the patient is responding to antidepressant treatment with some symptom reduction but has not yet reached remission”; and 3) a patient in the remission phase of the condition: “By remission phase, we mean the patient feels better and experiences a significant reduction in symptoms compared to the acute or post-acute phase. Some residual symptoms may persist but are significantly fewer in number and severity compared to other phases.” The phase (i.e., acute; post-acute; remission) definition was specified at the start of each relevant section of the interview, and respondents could remind themselves of the definition at any stage of the interview.

### Exclusion Criteria

Patients were excluded if they were employed by a pharmaceutical company or marketing agency, or if they were employed by an unsuitable entity, such as the government or a pharmacy, as such occupations may have biased their responses to research based on survey data. Patients were also precluded from taking part in the survey if they were younger than 18 years (all countries but the United States) or 25 years (United States only) (see **Supplementary Appendix B, Patient Questionnaire**).

Similar to patients, HCPs were excluded from participation in the survey if they were employed by a pharmaceutical or market research company, or if (in the United States only) they or an immediate family member was employed by an unsuitable agency, such as the FDA (see **Supplementary Appendix C, HCP Questionnaire**).

Patients and HCPs were excluded if they discontinued the survey for any reason. Finally, patients and HCPs were removed from the final sample if they were deemed to have 1) provided their answers to a series of questions in the same place on a rating scale, 2) completed the study in an unrealistically short time (i.e., less than 40% of the mean time taken by patients and HCPs), or 3) provided nonsensical open-ended responses.

### Assessment

Patients completed a 25-min online survey (**Supplementary Appendix B**) that required them to answer questions based mainly on their current experiences with MDD. Each patient self-selected the phase of depression (acute, post-acute, and remission; see **Supplementary Appendix A Table 1**) that they were experiencing based on the definitions given within the survey and were asked relevant questions accordingly.

HCPs completed a 30-min online survey (**Supplementary Appendix C**) that required them to complete three patient record forms, each one corresponding to the last patient they treated who was experiencing each of the three phases of depression, definitions of which were provided to HCPs throughout the survey and described in **Supplementary Appendix A Table 1**. HCPs were instructed to refer to patient records when completing the patient forms. Both patients and HCPs completed the Functioning Assessment Short Test (FAST) questionnaire [incorporated into the patient cohort questionnaire at Q22 (**Supplementary Appendix B**), and into the HCP questionnaire at Q27/Q58/Q89 (**Supplementary Appendix C**)]. In both patient and HCP versions of the FAST, the language was slightly amended from the original version to account for data collection *via* online survey. The FAST scale consists of 24 items developed for the clinical evaluation of the main difficulties in daily functioning presented by psychiatric patients (17, 18). While originally validated in bipolar disorder patients, the FAST has recently been applied to patients with MDD (17, 19). As part of the FAST questionnaire, each respondent was asked to select the degree of difficulty (“no difficulty,” “mild difficulty,” “moderate difficulty,” “severe difficulty,” or “don’t know”) associated with each of the 24 items relating to psychosocial functioning. All respondents who selected “don’t know” for five or more items were removed from the analysis of the FAST total scores (*N* = 62), but the rest of their questionnaire responses were retained for analysis.

To determine treatment priorities and goals held by patients and by HCPs for their patients, each group was asked to choose a single primary treatment goal from among several suggested [Patient Questionnaire Q20 (**Supplementary Appendix B**), HCP Questionnaire Q24/55/86 (**Supplementary Appendix C**)].

### Statistical Analysis

Summary statistics using mean, median, or percentages were estimated for demographic and clinical characteristics, symptoms experienced, FAST scores [with standard deviation (SD)], and treatment goals. Data are presented for the patient and HCP groups and include statistical tests, where relevant, between the cohorts (independent *z*-tests for proportions and independent *t*-tests with Bonferroni correction for means).

## Results

### Participant Characteristics

The size of the online panel of patients and HCPs per country is presented in **Table 1**, together with invitations sent and response rates and completion. From the initial 2,379 patient and 1,223 HCP respondents, 371 patient and 177 HCP respondents were removed due to failure to complete the survey or suspected inaccuracy (see the Exclusion Criteria section of Methods), leaving a total of 2,008 patients and 1,046 HCP respondents. Patients who answered “don’t know” to five or more items/statements from the FAST questionnaire (*N* = 62) were excluded from the FAST analysis, resulting in a total patient sample of 1,946 respondents. Of the 1,046 HCP respondents, 680 were specialists (650 psychiatrists and 30 neurologists), and 366 were PCPs. HCPs completed the FAST questionnaire for one of their patients in each of the three disease phases, resulting in forms for 3,138 unique patients. Responses from the acute, post-acute, and remission phases for 41, 29, and 26 patients, respectively, were excluded from analysis, resulting in 3,042 unique reports.

**Table 1:** Patient and HCP sample.

Country	Brazil	Canada	Mexico	South Korea	United States	France	Italy	Spain	Total
HCPs									
Panel size (psychiatrists)	2,200	620	520	600	4,200	1,380	850	980	11,350
Panel size (PCPs)	4,400	3,800	1,850	NA	4,800	4,650	1,560	1,750	22,810
Panel size (neurologists)	NA	NA	NA	NA	NA	NA	720	NA	720
Total panel size	6,600	4,420	2,370	600	9,000	6,030	3,130	2,730	34,880
Total sample*	376	431	282	207	363	281	290	198	2,428
Excluded prior to survey**	208	281	139	34	163	126	144	44	1,139
Excluded due to response***	39	21	15	26	22	24	17	13	117
Excluded due to max sample size per country****	1	0	0	22	26	4	2	11	66
Completers total	128	129	128	125	152	127	127	130	1,046
Patients									
Panel size	226,000	192,000	114,000	83,000	1,269,000	273,000	167,000	128,000	2,452,000
Total sample*	550	605	1,375	3,843	492	537	3,923	2,723	14,048
Excluded prior to survey**	248	217	824	3,148	190	172	2,895	1,635	9,329
Excluded due to response***	19	46	31	89	52	36	54	44	371
Excluded due to max sample size per country****	32	91	269	353	0	79	724	792	2,340
Completers total	251	251	251	253	250	250	250	252	2,008

*Total sample invited to participate in survey was selected by random selection among panel members. **Exclusion prior to survey included exclusion for any one of exclusion criteria. ***Exclusion due to response included: quit survey, provided their answers to a series of questions in the same place on a rating scale; completed the study in an unrealistically short time (i.e., less than 40% of the mean time taken by patients and HCPs); or provided nonsensical open-ended responses. ****A maximum requested sample was 250 patients per country (with a minimum of 50 patients in the acute, post-acute, and remission phase), and 125 HCPs per country. Patients and HCPs who signed up after maximum was reached were excluded from the survey.

HCPs, health care providers; NA, not available; PCPs, primary care physicians.

Patient- and HCP-reported demographic and baseline characteristics were comparable (**Table 2**): 61% of the patients were female, as were 64% of the patients assessed by HCPs. In both patient- and HCP-assessed cohorts, the majority of patients were older than 31 years, and 58% of patients reported >1 depressive episode. The majority of the patients (80%) reported receiving antidepressants or antidepressants plus psychological treatment (e.g., cognitive behavioral therapy) for MDD; HCPs reported that 96% of their patients were in these categories.

**Table 2:** Demographic and clinical characteristics and FAST scores.

	Patient cohort (*N* = 2,008)	HCP-assessed cohort (*N* = 3,138)
Males, n (%)	783 (39)	1,130 (36)
Females, n (%)	1,225 (61)	2,008 (64)
Mean age, years	45	44
18–30 years, n (%)	341 (17)	565 (18)
31–50 years, n (%)	904 (45)	1,569 (50)
≥51 years, n (%)	763 (38)	1,004 (32)
**Current treatments**		
Antidepressants only (no psychological therapy, e.g., CBT), n (%)	1,537 (77)	2,830 (90)
Antidepressants plus psychological therapy (e.g., CBT), n (%)	55 (3)	192 (6)
Psychological therapy (e.g., CBT) only (no antidepressants), n (%)	49 (2)	25 (1)
Other drug therapy, n (%)	153 (8)	67 (2)
No treatment or therapy prescribed, n (%)	–	24 (1)
Unsure/don’t know, n (%)	110 (5)	–
None of the above, n (%)	104 (5)	–
**Number of episodes among patients who had multiple episodes (*n* = 1,169)**		
Acute, mean (median)	6.83 (4.00)	N/A
Post-acute, mean (median)	4.91 (3.00)	N/A
Remission, mean (median)	4.15 (3.00)	N/A
**FAST total scores** ^a^^,^^b^		
Acute, n	406	1,005
Acute score, mean [SD] (median)	48.71 [13.72] (51)	47.75 [12.32] (49)
Post-acute, n	767	1,017
Post-acute score, mean [SD] (median)	43.36 [14.22] (46)	35.87 [13.80] (36)
Remission, n	773	1,020
Remission score, mean [SD] (median)	33.38 [16.23] (34)	22.68 [15.36] (22)
Overall FAST total score, mean (median)	40.51 (43)	38.45 (41)

Percentage data refer to proportion of patients.

a Q22 (Patient) To what extent are you experiencing difficulties with the following aspects during this phase of depression? Baseline: All patients excluding those who stated ‘don’t know’ to 5 or more statements: acute n = 406, post-acute n = 767, remission n = 773.

b Q27/58/89 (HCP) To what extent is the patient experiencing difficulties with the following aspects during this phase of depression? Baseline: All HCP patient case records excluding those who stated ‘don’t know’ to 5 or more statements: acute n = 1,005, post-acute n = 1,017, remission n = 1,020.

CBT, cognitive behavioral therapy; FAST, Functioning Assessment Short Test; HCP, health care professional; N/A, not applicable; N, total population; n, subset of population; SD, standard deviation.

### Symptoms Experienced

**Figure 1:**
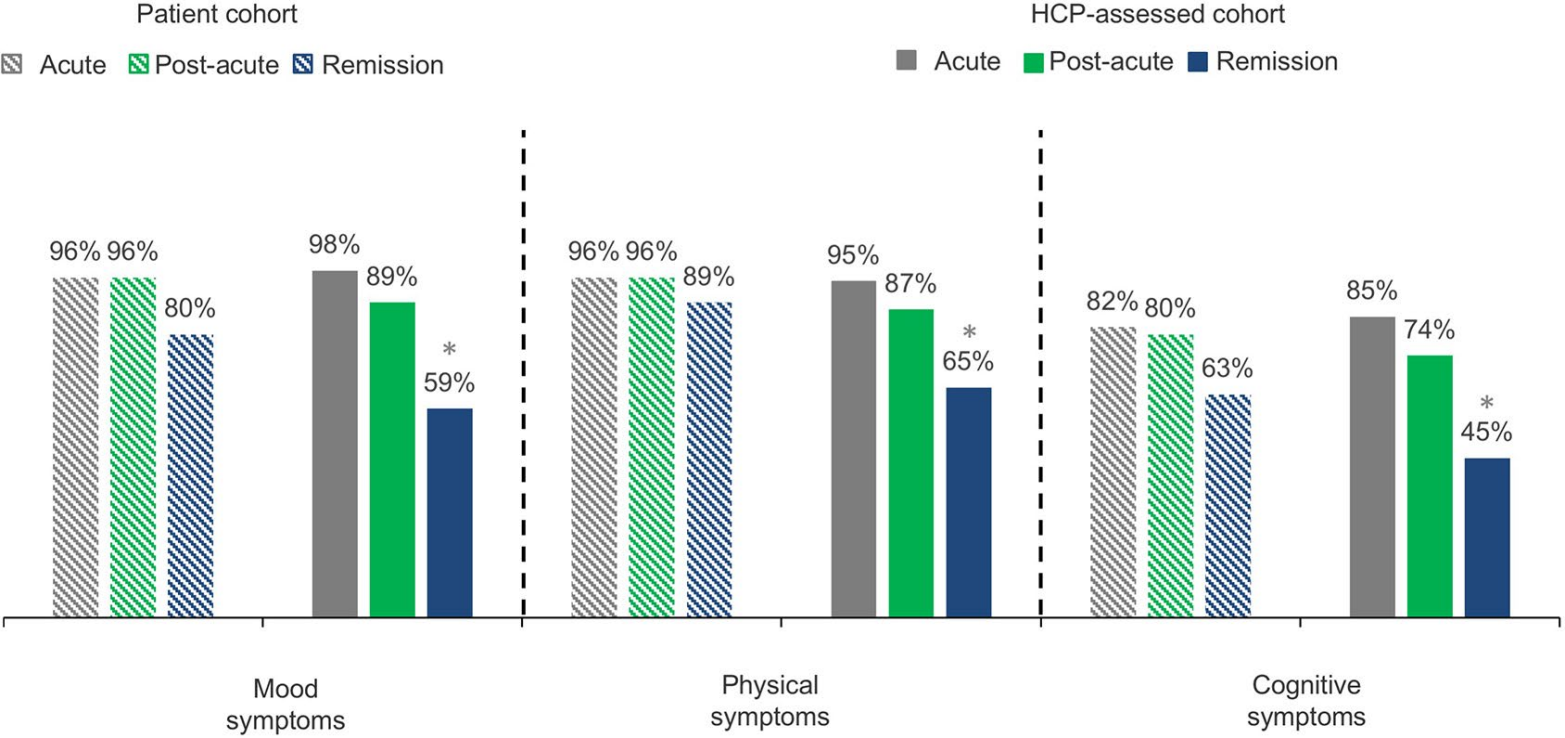
The percentage of patients from the patient cohort and the HCP-assessed cohort experiencing mood, physical, and cognitive symptoms. Percentage data refer to proportion of patients experiencing mood, physical, and cognitive symptoms. Q15 (Patient) Which symptoms are you currently experiencing during this phase of depression? Baseline: patients, acute n = 425; post-acute, n = 793; remission, n = 790. Q18/49/80 (HCP) Which symptoms did the patient experience during this phase of depression? Baseline: All HCP patient case records: acute, n = 1,046; post-acute, n = 1,046; remission, n = 1,046. HCP, health care professional; n, subset of population. *p < 0.0001.

A similar prevalence of symptoms was reported by patients and HCPs across domains (mood, physical, and cognitive) in the acute [mood (96% vs. 98%), physical (96% vs. 95%), cognitive (82% vs. 85%)], and the post-acute [mood (96% vs. 89%), physical (96% vs. 87%), cognitive (80% vs. 74%)] phases of MDD (**Figure 1**). Interestingly, in the remission phase of MDD, HCPs reported a significantly lower prevalence of symptoms across all three domains than the prevalence reported by the patient cohort [mood (59% vs. 80%, p < 0.0001), physical (65% vs. 89%, p < 0.0001), cognitive (45% vs. 63%, p < 0.0001)].

**Figure 2:**
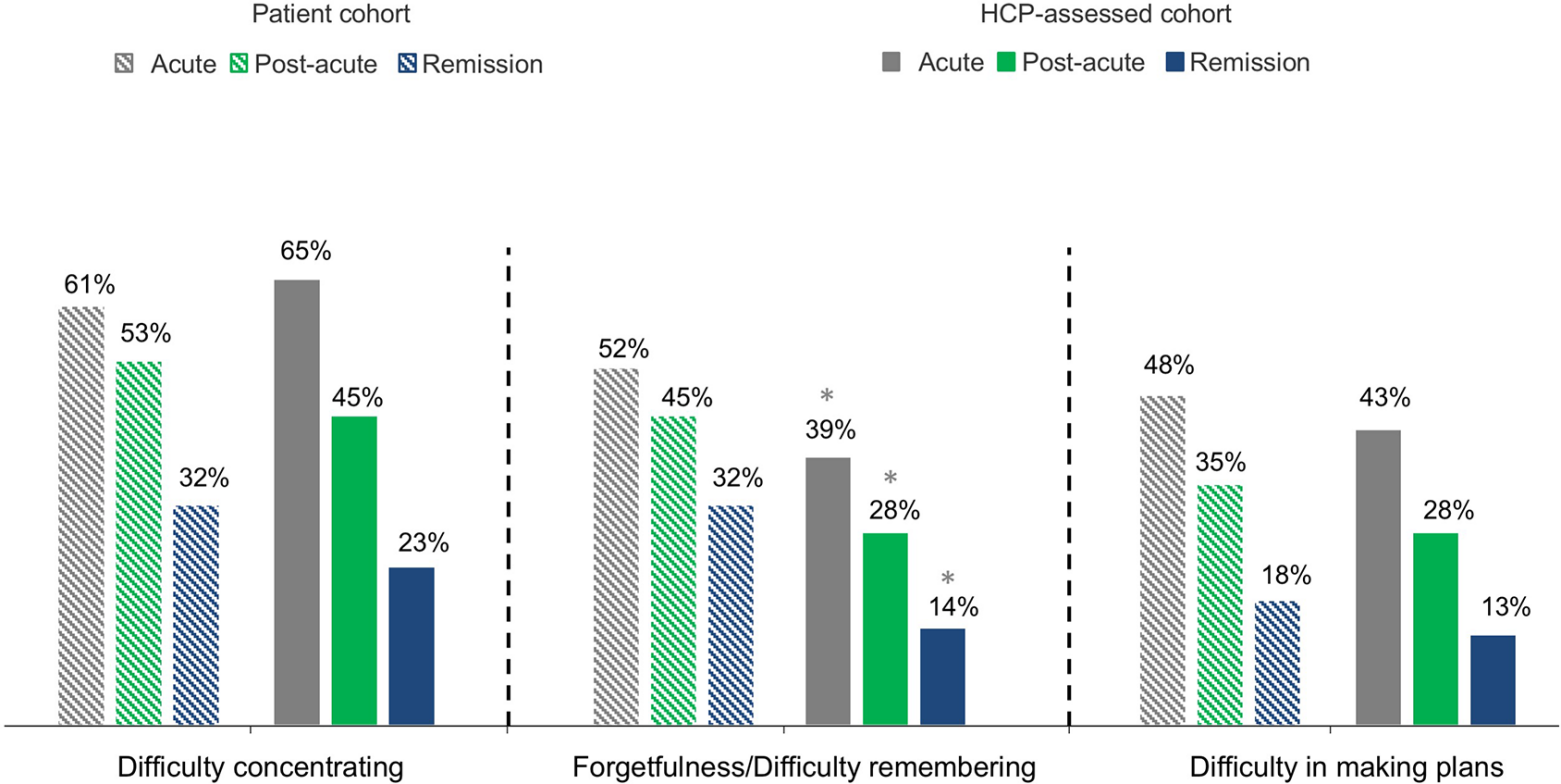
The percentage of all patients in the patient cohort and the HCP-assessed cohort experiencing cognitive symptoms at each phase. Percentage data refer to proportion of patients experiencing cognitive symptoms at each phase. Q15 (Patient) Which symptoms are you currently experiencing during this phase of depression? Baseline: patients, acute, n = 425; post-acute, n = 793; remission, n = 790. Q18/49/80 (HCP) Which symptoms did the patient experience during this phase of depression? Baseline: All HCP patient case records; acute, n = 1,046; post-acute, n = 1,046; remission, n = 1,046. HCP, health care professional; n, subset of population. *p < 0.0001.

Regarding specific cognitive symptoms, a similar proportion of patients and HCPs reported difficulty concentrating (61% vs. 65%) and difficulty making plans (48% vs. 43%) in the acute phase, while fewer HCPs reported forgetfulness/difficulty remembering (39% vs. 52%, p < 0.0001) as being a problem in their MDD patients (**Figure 2**). In the post-acute and remission phases, HCPs reported that a significantly lower percentage of patients had these specific cognitive symptoms, in particular forgetfulness/difficulty remembering, compared with the patient cohort’s reports [(28% vs. 45%, p < 0.0001) and (14% vs. 32%, p < 0.0001), respectively] (**Figure 2**).

Regarding perception of severe symptoms of MDD, respondents from both groups confirmed the presence of high-severity symptoms in mood, physical, and cognitive domains of depression during the acute phase. However, in the post-acute and remission phases, significantly fewer HCPs reported any severe symptoms as compared with patients [post-acute (28% vs. 66%, p < 0.0001); remission (10% vs. 28%, p < 0.0001), respectively].

### Psychosocial Functioning

The impact of depression on psychosocial functioning, as measured by the total FAST score, is reported in **Table 2**. In the acute phase, the mean total FAST score (48.71 vs. 47.75) and subdomain scores were comparable between the patient- and HCP-assessed cohorts. In contrast, in the post-acute (43.36 vs. 35.87, p < 0.0001) and remission (33.38 vs. 22.68, p < 0.0001) phases, patients reported significantly more impairment of psychosocial functioning than the HCP-assessed cohort (**Table 2**). In the post-acute phase, a greater percentage of patients reported severe difficulties (above the median value) for autonomy (51% vs. 41%, p < 0.0001), cognitive functioning (51% vs. 43%, p = 0.0008), financial issues (46% vs. 34%, p < 0.0001), interpersonal relationships (56% vs. 48%, p = 0.0008), and leisure time (50% vs. 28%, p < 0.0001); a similar pattern was also seen during the remission phase.

**Figure 3:**
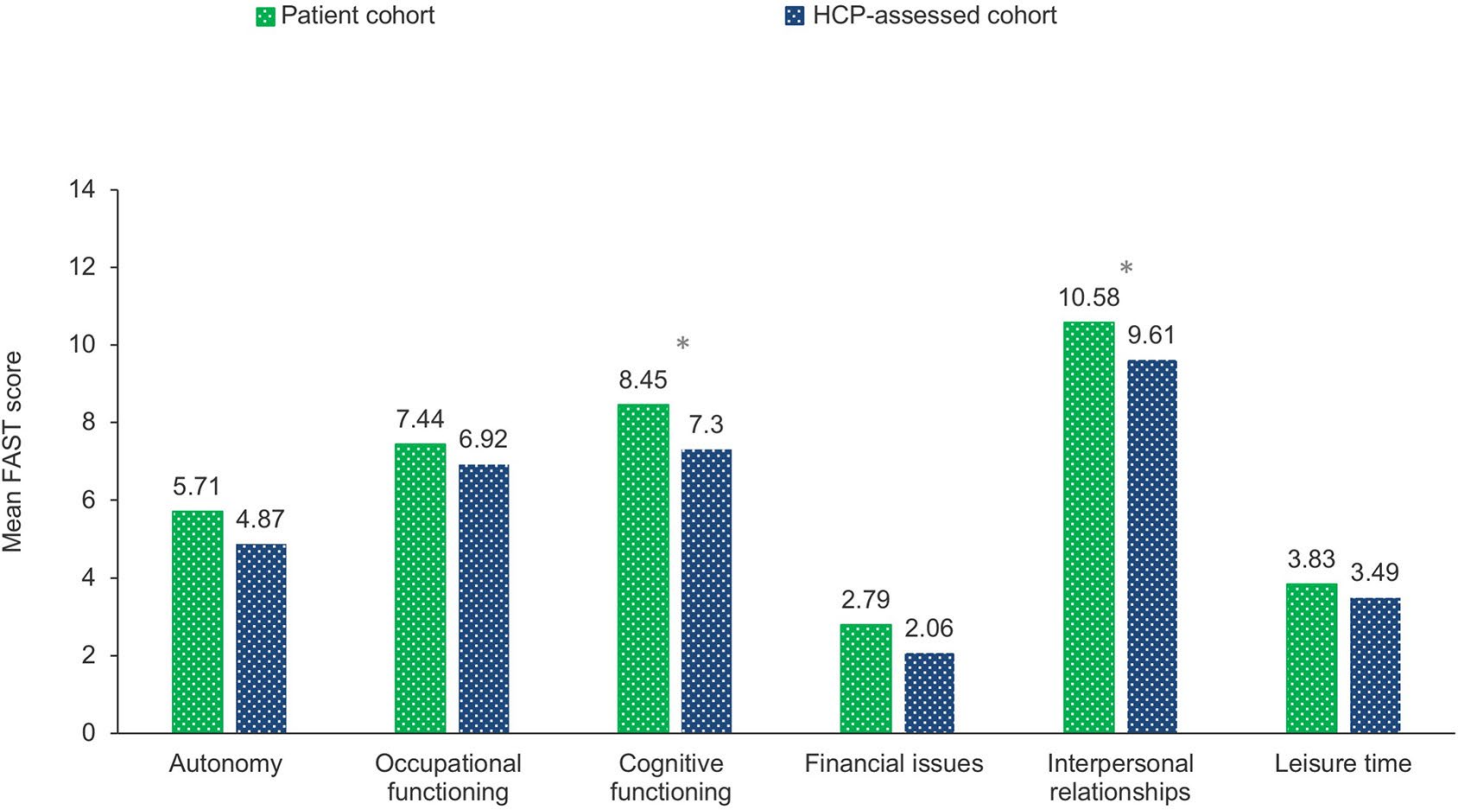
The mean FAST score as a sum of all phases by patient cohort and HCP-assessed cohort for each domain described in the questionnaire. Q22 (Patient) To what extent are you experiencing difficulties with the following aspects during this phase of depression? Q27/58/89 (HCP) To what extent is the patient experiencing difficulties? Baseline: All patients/HCPs excluding those who stated “don’t know” to more than 20% of responses within each of the respective domains. FAST, Functioning Assessment Short Test; HCP, health care professional; n, subset of population. *p < 0.0001.

When considering the FAST score as a sum across all phases, the patient cohort had higher FAST scores than did the HCP-assessed cohort for all functional domains (**Figure 3**), indicating generally greater psychosocial impairment from the patients’ perspective. The largest differences in FAST score between patients and HCPs were observed in cognitive functioning (mean = 8.45 vs. 7.30, respectively; p < 0.0001) and interpersonal relationships (mean = 10.58 vs. 9.61, respectively; p < 0.0001).

For functional domains of the FAST and phase of MDD, the differences in FAST scores were greatest in the post-acute phase for the cognitive functioning domain (8.85 vs. 7.32 in patient- and HCP-assessed cohort, respectively; p < 0.0001) and in the remission phase for the cognitive functioning domain (7.14 vs. 4.88 in patient- and HCP-assessed cohorts, respectively; p < 0.0001) (**Supplementary Appendix D, Figures 1 and 2**).

### Treatment Goals

Symptom relief was given a higher priority by the patient cohort across all symptom domains and phases of depression than that by the HCPs. In the acute phase, 92% of patients vs. 88% of HCPs wanted mood (p = 0.0281), 84% vs. 64% wanted physical (p < 0.0001), and 71% vs. 39% (p <0.0001) wanted cognitive symptoms addressed (**Table 3**).

**Table 3:** Patient- and HCP-assessed symptoms that need to be addressed by treatment and those that are not adequately treated.

**Domain**	**Phase**	Symptoms most needed to be addressed by treatment		Symptoms not adequately treated	
		^a^Patient cohort, %	^b^HCP-assessed cohort, %	^c^Patient cohort, %	^d^HCP-assessed cohort, %
Mood	Acute	92*	88	75**	47
	Post-acute	91**	75	69**	51
	Remission	83**	64	60**	43
Physical	Acute	84**	64	72**	36
	Post-acute	79**	58	66**	39
	Remission	74**	62	65**	40
Cognitive	Acute	71**	39	55**	33
	Post-acute	58**	47	49**	32
	Remission	56	49	40	40

Data are percentage of patients.

a Q19 (Patient) What are the symptoms that you most want your treatment to address? Baseline: Patients who experience each group of symptoms. Baseline: patients, mood: acute, n = 408; post-acute, n = 761; remission, n = 635/physical: acute, n = 407; post-acute, n = 761; remission, n = 701/cognitive: acute, n = 347; post-acute, n = 636; remission, n = 500.

b Q26/57/88 (HCP) What symptoms does the patient most want addressed with the treatment prescribed? Baseline: PRFs who experience each group of symptoms. Baseline: HCP PRFs mood: acute n = 1,023, post-acute n = 929, remission n = 614/physical: acute n = 994, post-acute n = 912, remission n = 683/cognitive: acute n = 894, post-acute n = 769, remission n = 467.

c Q21 (Patient) Which symptoms, if any, do you feel your current treatment does not adequately treat during this phase of depression? Baseline: Patients who experience each group of symptoms. Baseline: patients, mood: acute, n = 408; post-acute, n = 761; remission, n = 635/physical: acute, n = 407; post-acute, n = 761; remission n = 701/cognitive: acute, n = 347; post-acute, n = 636; remission, n = 500.

d Q25/56/87 (HCP) Which of the following symptoms that this patient has experienced during this phase of depression can you NOT adequately treat? Baseline: PRFs who experience each group of symptoms. Baseline: HCP PRFs, mood: acute n = 1,023, post-acute n = 929, remission, n = 614/physical: acute, n = 994; post-acute n = 912; remission, n = 683/cognition: acute n = 894; post-acute, n = 769; remission, n = 467.

HCP, health care professional; n, subset of population; PRF, patient record form.

z-tests undertaken on patient and HCP-assessed cohorts to determine statistically significant differences:

*The difference between patient and HCP cohort is statistically significant at the 95% confidence interval, **The difference between patient and HCP cohort is statistically significant at the 99% confidence interval.

Significant differences between the patient- and HCP-assessed cohorts were observed as to how adequately symptoms were treated across the different domains and phases of depression, except cognitive symptoms in the patients who were in remission. Overall, HCPs reported a lower proportion of patients being inadequately treated for mood (1.4- to 1.6-fold difference in magnitude), physical (1.6- to 2.0-fold difference in magnitude), and cognitive (0- to 1.8-fold difference in magnitude) symptoms across all three phases of depression. While the proportion of patients reporting inadequate treatment decreased from the acute to the remission phase for all symptom domains, the proportion of HCPs reporting inadequate treatment was quite stable across symptom domains and phases. For mood symptoms, 43% of the HCPs found these inadequately treated in patients they considered to be in remission.

**Figure 4:**
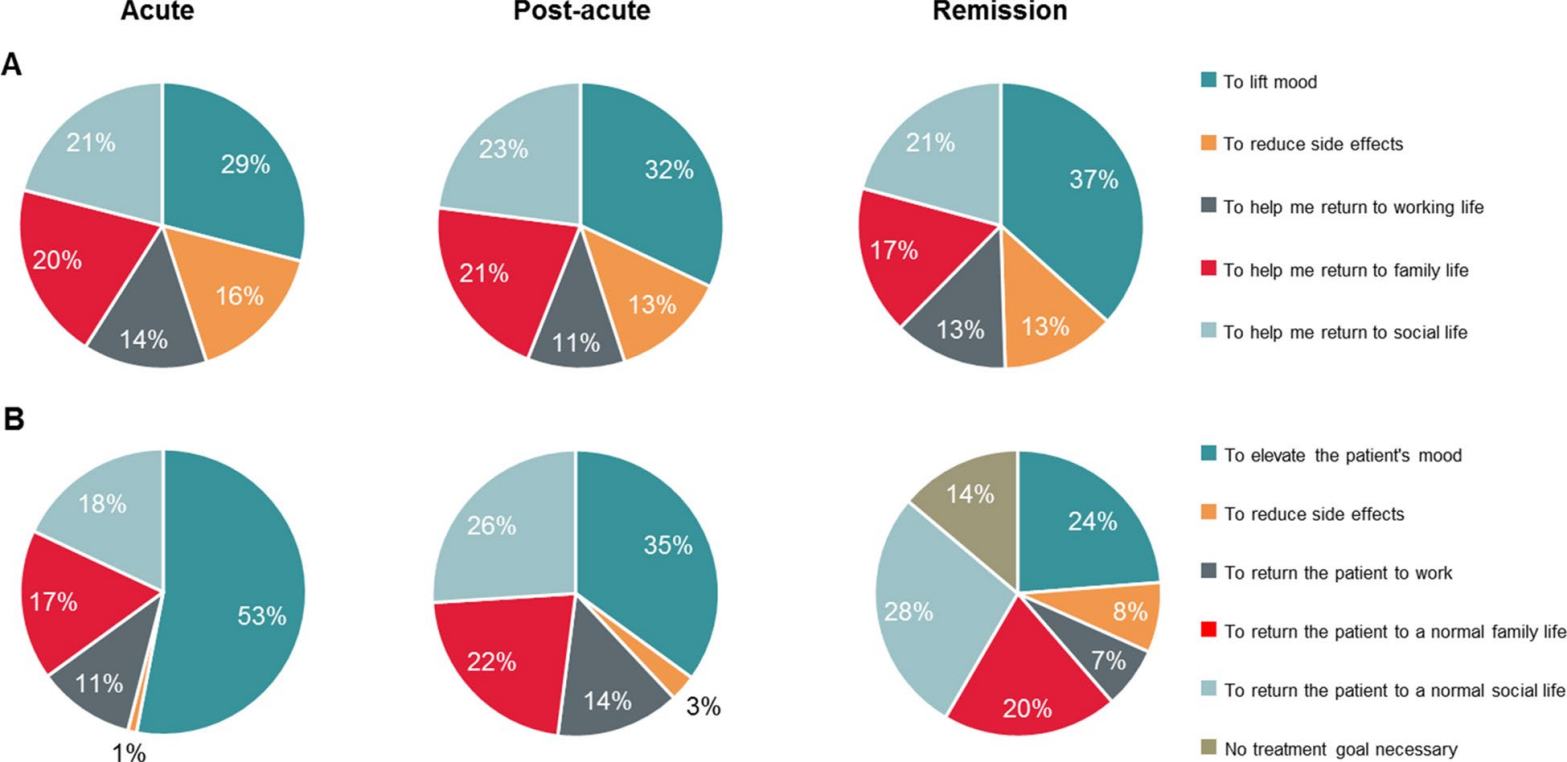
**(A)** Treatment goals for patient cohort in each phase. **(B)** HCP-assessed cohort’s treatment goals. Percentage data refer to the proportion of patients. Q20 (Patient) Which ONE of the following best reflects your hopes for treatment during this phase of depression? Baseline: patients, acute n = 425; post-acute, n = 793; remission, n = 790. Q24/55/86 (HCP) Which ONE of the following best reflects your primary treatment goal during this phase of depression? Baseline: All HCP patient case records. Acute, n = 1,046; post-acute, n = 1,046; remission, n = 1,046. Because of rounding, percentages may not add up to 100%. HCP, health care professional; n, subset of population.

The patient cohort tended to report similar goals for treatment (“to elevate my mood,” “to help me return to normal family life,” “to reduce side effects,” “to help me return to a normal social life,” and “to help me return to work”) across the different phases of depression (**Figure 4A**). In contrast, HCPs’ goals for their patients changed by phase of MDD; mood improvement was the primary goal for patients in the acute phase, whereas the primary goal was the patients’ return to normal functioning (family, social, and work) in the post-acute and remission phases (**Figure 4B**).

## Discussion

This is the first study to examine similarities and differences in perceptions of MDD symptoms and treatment priorities between patients and HCPs across the acute, post-acute, and remission phases of the disease. We intended to better understand the symptoms that patients experience and most want treated, to identify the impact on patients’ psychosocial functioning, and to ascertain patients’ and HCPs’ treatment priorities. While direct comparisons between the patient and HCP reports should be done with caution, we found that patients reported more mood, physical, and cognitive symptoms than HCPs in the post-acute and remission phases of MDD. We also found that HCPs estimated less impact of MDD on psychosocial functioning compared with the patients’ estimations, particularly in the post-acute and remission phases. Addressing mood symptoms was a priority for both patients and HCPs in the acute phase of MDD; however, patients also gave high emphasis to addressing physical and cognitive symptoms at this stage of the disease as well as the impact of MDD on psychosocial functioning. In our survey, we observed a substantial difference in the perception of the effectiveness of treating symptoms of MDD between patients and HCPs.

The observation that HCPs report fewer symptoms in the post-acute and remission phases is critical, as several studies have identified a correlation between the presence of residual symptoms and an increased risk of relapse (16, 20, 21). Furthermore, patients experiencing residual symptoms are likely to have a more chronic course of the disease and higher recurrence rates in comparison to MDD patients in remission who are asymptomatic (22–24).

In our study, cognitive symptoms were persistent and highly prevalent in the post-acute and remission phases as reported by patients, and, to a lesser extent, by HCPs. This finding is consistent with the results of several studies reporting persistence of cognitive symptoms in the remission phase (12, 25). The lower reporting of cognitive symptoms by HCPs in the remission phase could indicate less awareness and/or attention to these symptoms, as previously suggested by a survey of PCPs and psychiatrists in the United Kingdom (26). As cognitive symptoms of MDD are associated with psychosocial and functional impairment, low quality of life (4), and a higher risk of relapse (16), it is important that such symptoms are identified and managed.

Closely related to symptom perception is treatment goal setting. Regarding overall treatment goals, our research revealed interesting differences between patients and HCPs, in particular the very prominent focus on lifting mood in the acute phase among HCPs, and the consistent emphasis on both mood and functioning among patients throughout the phases of MDD. This finding is in line with earlier research that showed an emphasis among physicians to treat to symptom resolution (6), while patients emphasized restoring functioning, such as a return to normal family and social life and return to work (8, 9).

The finding that HCPs reported seeing improved psychosocial functioning (FAST score) with patients in the remission phase, while patients experienced a more consistent functional impairment across phases, underscores the different perceptions of the degree of psychosocial impairment between patients and HCPs, especially during remission. An increased focus on psychosocial function during treatment planning may be warranted, including a thorough assessment of the patient’s perspective. If both significant symptoms as well as functional impairment do persist into the post-acute and remission stages of MDD, it could suggest the need to modify pharmacological or non-pharmacological treatments to reach better symptomatic and functional outcomes. Recent guidelines advocate for the ongoing evaluation of the patients’ psychosocial functional impairment and quality of life, including their occupational, social, and educational functioning (27). Moreover, the American Psychiatric Association recommends that the treatment goal for the acute phase of MDD should be achieving remission and a return to full psychosocial functioning and quality of life (28).

Given the observed differences between patients and HCPs, it is worthwhile to re-consider the clinical value of improved alignment between patient and HCP perceptions on symptoms, functioning, and treatment goals. A patient-centered treatment strategy has been advocated for several years (29). Improvements in depressive symptoms in patients with MDD have been reported with patient-centered consulting approaches, such as formal consideration of the patient’s needs and priorities, development of treatment plans, and patient-centered assessment of cognitive symptoms using assessment tools (30). While the goals of many HCPs are focused on improvement of symptoms, our study suggests that a focus beyond symptoms onto patients’ quality of life and psychosocial functional goals may be warranted. A possible strategy to better align HCPs’ and patients’ perceptions and goals is the use of collaborative care programs, including measurement-based care, treat-to-target approaches, and stepped care in which treatments are systematically adjusted (31). Overall, higher continuity of care has been linked to lower mortality for patients suffering from MDD (32).

Several limitations of our research should be noted. First, the patient- and HCP-provided responses were unmatched, limiting the analysis of our data mainly to within-group comparisons and comparisons of overall patterns between HCP and patient data. Second, a selection bias may have been present in patients who volunteered for the survey; respondents may have been more opinionated about their care and therefore may not represent the broader MDD patient population. Similarly, the HCPs who chose to respond may have had strong opinions about how their patients with MDD experience symptoms and their treatment priorities. Third, information about comorbid psychiatric disorders and the presence of subthreshold symptoms was not collected, and, as such, results at this level of granularity could not be provided. Fourth, this was an Internet-based study, which may have affected the way in which both patients and HCPs responded. It is a common perception that face-to-face interviews are optimal for obtaining accurate patient responses; however, such studies are time-consuming and, therefore, often small in size. The use of an Internet-based survey in this study allowed us to obtain a large sample size in a relatively short period, enabling detection of cross-sectional response patterns between these two cohorts. Fifth, we included patients and HCPs from eight countries, introducing the possibility of culturally based differences in perception of symptoms, the analysis of which is beyond the scope of this manuscript. Finally, given that three types of HCPs participated in the study, possible between-group differences among the HCPs were not accounted for in this analysis, but could be an interesting focus of future research.

## Conclusions

Patients with MDD reported more mood, physical, and cognitive symptoms than HCPs in the post-acute and remission phases of MDD, as well as a greater impact on psychosocial functioning. Patients more frequently reported inadequately treated symptoms across the domains and phases of MDD. Addressing mood symptoms was a priority for both patients and HCPs in the acute phase of MDD; however, patients also emphasized the need for improvement in physical and cognitive symptoms as well as the need to address the impact of MDD on psychosocial functioning. Given the observed discordance between patient and HCP perceptions, a stronger emphasis on patient-centered care and shared decision making processes between HCPs and patients may be warranted to treat symptoms and functioning in the different stages of MDD to reduce residual symptoms and to formulate treatment goals that are shared between patients and HCPs. A better alignment on perceptions, goals, and expectations may enable a stronger focus on psychosocial functioning overall and earlier in the treatment course and improve longer-term outcomes in MDD.

## Ethics Statement

All panel members (both patients and HCPs) accepted the online panel partners privacy policies and terms and conditions when they signed up to become a member of the panel. They thus gave their consent to receive invitations to participate in market research and, further, their consent was sought again to participate in this particular study. The market research protocol was not formally approved by a medical ethics committee.

## Author Contributions

MC and BB were both instrumental in the development of the study, study design, analysis plan, and interpretation of data and writing of the manuscript.

## Funding

This study was funded by H. Lundbeck A/S, who contributed to the data analysis, review of the data, and review of the manuscript.

## Conflicts of Interest Statement

BB received speaker/consultation fees from AstraZeneca, Lundbeck, Pfizer, Takeda, Servier, Bristol-Myers Squibb, Otsuka, and Janssen-Cilag. MC is an employee of Lundbeck A/S.
