# Supplementary material for: Differences in Perceptions of Major Depressive Disorder Symptoms and Treatment Priorities Between Patients and Health Care Providers Across the Acute, Post-Acute, and Remission Phases of Depression
Source: Front Psychiatry. 2019 May 21;10:335. doi: 10.3389/fpsyt.2019.00335 (PMC6537882; doi:10.3389/fpsyt.2019.00335)
Supplement: Supplementary file 1 [file Table_2.docx]

**Supplementary appendix A**

| **Supplementary Table 1. Definitions used for phases of depression** | |
| --- | --- |
| **Phase** | **Description** |
| Acute | A patient experiencing acute symptoms of major depressive disorder that require antidepressant treatment |
| Post-acute | A patient experiencing a phase where the patient is responding to antidepressant treatment with some symptom reduction but has not yet reached remission |
| Remission | A patient who feels better and experiences a significant reduction in symptoms compared with the acute or post-acute phase. Some residual symptoms may persist but are significantly fewer in number and severity than in other phases |

**Supplementary appendix B**

**Patient questionnaire**

**PR 0072 MDD HCP and Patient Journey Research: Patient Questionnaire**

**<PN: PLEASE ADD IN STANDARD QC QUESTIONS AS NECESSARY>**

**Section 1: Introduction and screening (5 minutes)**

Thank you for agreeing to take part in this research, this survey is conducted by a company called BPR on behalf of a pharmaceutical company and will focus on the area of depression. The survey should take approximately 25 minutes to complete and all of your personal details will remain confidential and will not be divulged or passed onto the client or any other organisation. The aim of the research is to understand the experiences of people with depression so that potential improvements can be made to future treatments.

Before we begin, we need to ask you a short series of questions to check your suitability for the survey.

**Adverse event reporting**

Different patients sometimes respond in different ways to the same medicine, and some side effects may not be discovered until many people have used a medicine over a period of time. For this reason, we are now required to pass on to our client, details of any side effects or complaints related to their own products that are mentioned during the course of market research. Although what you say will, of course, be treated in confidence, should you mention during the survey a side effect when you, or someone you know, became ill after taking one of our client’s medicines, or a problem you have had with one of our clients medicines, we will need to report this, so that they can learn more about the safety of their medicines.

S1. Please confirm you are happy to continue on this basis?

**<PN: SINGLE CODE> <PN: ASK ALL RESPONDENTS>**

| Yes | 1 | **Continue** |
| --- | --- | --- |
| No | 2 | **Screen out** |

S2. Have you ever been diagnosed with depression by a doctor?

**<PN: SINGLE CODE> <PN: ASK ALL RESPONDENTS>**

| Yes | 1 | **Continue** |
| --- | --- | --- |
| No | 2 | **Screen out** |

S3. Are you currently using a medication to treat depression that has been prescribed by a doctor?

**<PN: SINGLE CODE> <PN: ASK ALL RESPONDENTS>**

| Yes | 1 | **Continue to S5** |
| --- | --- | --- |
| No | 2 | **Continue to S4** |

S4. Have you used a medication to treat depression that has been prescribed by a doctor **in the past 3 months**?

**<PN: SINGLE CODE> <PN: ASK ALL RESPONDENTS WHO CODE 2 AT S3>**

| Yes | 1 | **Continue to S5** |
| --- | --- | --- |
| No | 2 | **Thank and close** |

S5. The list below provides a description of the different phases of depression. Please carefully review the descriptions and indicate which one best describes the phase you currently experiencing:

**<PN: SINGLE CODE> <PN: ASK ALL RESPONDENTS>**

| **Acute** phase: a time when your symptoms are at their worst or most severe and for which you use antidepressant treatment | 1 | **Continue, code as ACUTE** |
| --- | --- | --- |
| **Post-Acute** phase: a time when your symptoms are starting to improve, are less severe than they were before and you still use antidepressant treatment | 2 | **Continue, code as POST-ACUTE** |
| **Remission** phase: a time when your symptoms have improved significantly and you are already feeling better, but you may or may not still experience some minor symptoms. You may or may not be using antidepressant treatment | 3 | **Continue, code as REMISSION** |

**<PN: IF A RESPONDENT WHO HAS CODED 1 AT S4 SELECTS A PHASE OF DEPRESSION OTHER THAN REMISSION (CODE 1 OR 2 AT S5) THEN THANK AND CLOSE>**

S6. A) Which if any of the other phases of depression have you experienced in **the past 3 months**?

**<PN: SINGLE CODE> <PN: ASK ALL RESPONDENTS> <PN: ONLY SHOW PHASES NOT SELECTED AT S5>**

| **Acute** phase: a time when your symptoms are at their worst or most severe and for which you use antidepressant treatment | 1 |
| --- | --- |
| **Post-Acute** phase: a time when your symptoms are starting to improve, are less severe than they were before and you still use antidepressant treatment | 2 |
| **Remission** phase: a time when your symptoms have improved significantly and you are already feeling better, but you may or may not still experience some minor symptoms. You may or may not be using antidepressant treatment | 3 |

B) And which phase did you experience most recently?

**<PN: SINGLE CODE> <PN: ASK ALL RESPONDENTS WHO CODE 2 PHASES AT S6A– ONLY SHOW CODES SELECTED>**

| **ACUTE** | **POST-ACUTE** | **REMISSION** |
| --- | --- | --- |
| 1 | 1 | 1 |

S7a. Are you currently employed by a pharmaceutical company or a market research agency?

**<PN: SINGLE CODE> <PN: ASK ALL RESPONDENTS>**

| Yes | 1 | **Screen out** |
| --- | --- | --- |
| No | 2 | **Continue** |

**<PN: ASK S7B ONLY IN US>**

S7b. Are you currently employed by or affiliated with any of the following?

**<PN: SINGLE CODE> <PN: ASK ALL RESPONDENTS>**

| An advertising agency or marketing research company | 1 | **Screen out** |
| --- | --- | --- |
| A company that manufactures, distributes, or sells pharmaceuticals or health care products | 2 | **Screen out** |
| A pharmacy or drug store | 3 | **Screen out** |
| The Food and Drug Administration (FDA) | 4 | **Screen out** |
| An HMO or health insurance company | 5 | **Screen out** |
| A doctor’s office, medical office or clinic | 6 | **Screen out** |
| None of the above | 7 | **Continue** |

S8. How old are you?

**<PN: NUMERIC 18-90> <PN: ASK ALL RESPONDENTS>**

**<PN: NUMERIC 25-70 in US>**

|  |  | Years |
| --- | --- | --- |

**Section 2: Background to patient (5 minutes)**

Thank you very much for taking part in this survey. We understand the subject is a very personal and sensitive one but we would ask you to please be as open and honest as you can be.

Across the next series of questions we would like to understand a little more about you and your experiences with depression so far.

1. Please indicate your gender:

**<PN: SINGLE CODE> <PN: ASK ALL RESPONDENTS>**

| Male | 1 |
| --- | --- |
| Female | 2 |

1. Which of the following best describes your highest level of education? <**PN: SINGLE CODE> <PN: ASK ALL RESPONDENTS>**

| Post-graduate | 1 |
| --- | --- |
| Undergraduate | 2 |
| High School Graduate | 3 |
| Vocational Qualification | 4 |
| No qualifications | 5 |
| Other | 6 |
| Don’t know | 7 |

1. Which of the following best describes your current working status? <**PN: SINGLE CODE> <PN: ASK ALL RESPONDENTS>**

| Full-time (30 or more hours per week) | 1 | **Go to Q3** |
| --- | --- | --- |
| Part-time | 2 | **Go to Q3** |
| Retired | 3 | **Go to Q4** |
| Homemaker/ Stay-at-home parent | 4 | **Go to Q4** |
| Student | 5 | **Go to Q4** |
| Unemployed | 6 | **Go to Q4** |

1. Please indicate which one of the following occupational groups best reflects the type of job you do:

<**PN: SINGLE CODE> <PN: ASK ALL RESPONDENTS>**

| **Semi or unskilled manual work** (e.g. Manual workers, apprentices to be skilled trades, Shop assistants etc.) | 1 |
| --- | --- |
| **Skilled manual worker** (e.g. Plumber, Lorry driver, Mechanic etc.) | 2 |
| **Supervisory or clerical** junior managerial/administrative (e.g. Office worker, Student Doctor, Foreman etc.) | 3 |
| **Intermediate managerial/ professional/ administrative** (e.g. Newly qualified doctor, Solicitor, Middle manager in large organisation) | 4 |
| **Higher managerial/ professional/ administrative** (e.g. Established doctor, Solicitor, Board Director in a large organisation, top level civil servant) | 5 |
| **Student** | 6 |
| **Casual worker** (not in permanent employment) | 7 |
| **Homemaker/ stay at home parent** | 8 |
| **Retired** | 9 |
| **Unemployed or not working due to long-term sickness** | 10 |
| **Other** | 11 |

1. Approximately how long ago were you first diagnosed with depression?

**<PN: NUMERIC. RANGE 0-90> <PN: ASK ALL RESPONDENTS>**

|  |  | Years |  |  |  | Months |  |  | Don’t know |
| --- | --- | --- | --- | --- | --- | --- | --- | --- | --- |

**Section 3: Current phase in journey (10 minutes)**

1. You previously mentioned that you are currently experiencing **<PN: INSERT PHASE ANSWER FROM S5>** phase of depression. For approximately how long have you been experiencing this current phase?

**<PN: NUMERIC> <PN: ASK ALL RESPONDENTS>**

|  |  | Weeks |  |  |  | Months |  |  | Years |
| --- | --- | --- | --- | --- | --- | --- | --- | --- | --- |

| Unsure / don’t remember | 1 |
| --- | --- |

1. What type of doctor do you currently see for your depression (and is responsible for prescribing your anti-depressant medication, if you are using one)?

**<PN: MULTI CODE> <PN: ASK ALL RESPONDENTS>**

| Primary care physician / family doctor | 1 |
| --- | --- |
| Psychiatrist | 2 |
| Neurologist | 3 |
| Geriatrician | 4 |
| Internal Medicine Specialist | 5 |
| Another specialist doctor | 6 |
| Other | 7 |
| Don’t know | 8 |

1. How long ago did you last visit this doctor **<PN: INSERT FROM Q7>** specifically about depression?

**<PN: SINGLE CODE> <PN: ASK ALL RESPONDENTS>**

|  |  | Weeks |  |  | Months |  |  |  | Years |
| --- | --- | --- | --- | --- | --- | --- | --- | --- | --- |

1. For approximately how long have you been seeing this doctor about your depression?

**<PN: SINGLE CODE> <PN: ASK ALL RESPONDENTS>**

|  |  | Weeks |  |  | Months |  |  |  | Years |
| --- | --- | --- | --- | --- | --- | --- | --- | --- | --- |

1. Have you always seen this doctor during this **<PN: INSERT PHASE>** of your depression?

**<PN: SINGLE CODE> <PN: ASK ALL RESPONDENTS>**

| Yes – I have only seen this doctor during this **<PN: INSERT PHASE>** | 1 |
| --- | --- |
| No - I have previously seen another doctor during this **<PN: INSERT PHASE>** | 2 |

1. What other type of doctors have you seen during this **<PN: INSERT PHASE>** of your depression?

**<PN: SINGLE CODE> <PN: ASK ALL RESPONDENTS>**

| Primary care physician / family doctor | 1 |
| --- | --- |
| Psychiatrist | 2 |
| Neurologist | 3 |
| Geriatrician | 4 |
| Internal Medicine Specialist | 5 |
| Another specialist doctor | 6 |
| Other | 7 |
| Don’t know | 8 |

1. Is this the first time in your life you have experienced this **<PN: INSERT PHASE>** of depression or have you experienced this **<PN: INSERT PHASE>** of depression before?

**<PN: SINGLE CODE> <PN: ASK ALL RESPONDENTS>**

| It’s the first time I’ve experienced this **<PN: INSERT PHASE>** phase | 1 | Go to Q14 |
| --- | --- | --- |
| I have experienced this **<PN: INSERT PHASE>** phase before | 2 | Go to Q13 |

1. Approximately, on how many separate occasions have you experienced this **<PN: INSERT PHASE>** of depression before?

**<PN: SINGLE CODE> <PN: ASK ALL RESPONDENTS WHO CODE 2 AT Q12>**

|  |  | Occasions |
| --- | --- | --- |

| Don’t know | 1 |
| --- | --- |

1. Has your working status changed because of this **<PN: INSERT PHASE>** phase of depression?

<**PN: MUTLI CODE> <PN: ASK ALL RESPONDENTS>**

| **Yes** – I have had to **reduce my working hours** | 1 |
| --- | --- |
| **Yes** – I have had to **take a** **less demanding job** | 2 |
| **Yes** – I have had to **take sick leave** | 3 |
| **Yes** – I have had to **stop working** | 4 |
| **No** – my working status **has not changed** | 5 |
| Don’t know | 6 |

1. Which of the following symptoms of depression are you currently experiencing during this **<PN: INSERT PHASE>**?

**<PN: MULTI CODE> <PN: ASK ALL RESPONDENTS> <PN: ROTATE ORDER IN WHICH ‘GROUPS’ OF SYMPTOMS SHOWN AND ORDER OF INDIVIDUAL SYMPTOMS WITHIN GROUPS>**

| Feeling sad / Low | 1 |
| --- | --- |
| Lack of energy | 2 |
| Lack of interest in general | 3 |
| Unable to take pleasure in things | 4 |
| Low self esteem | 5 |
| Lacking in confidence | 6 |
| Feelings of guilt or worthlessness | 7 |
| Suicidal thoughts | 8 |
|  |  |
| Reduced activity | 9 |
| Difficulty sleeping | 10 |
| Feeling tired | 11 |
| Waking early in the morning | 12 |
| Feeling agitated / Restless | 13 |
| Weight loss | 14 |
| Loss of sex drive | 15 |
| Loss of appetite | 16 |
| Depression worst in the morning | 17 |
|  |  |
| Forgetfulness / difficulty remembering | 18 |
| Slowness of thinking | 19 |
| Slowness in physical movements | 20 |
| Difficulty concentrating | 21 |
| Difficulty to prioritise / make decisions | 22 |
| Difficulty in making plans | 23 |
|  |  |
| Other (please specify) **<PN: ANCHOR>** | 24 |
| None of these symptoms **<PN: ANCHOR>** | 25 |

1. Overall, how severe would you rate the symptoms that you are experiencing during in this **<PN: INSERT PHASE>** phase?

**<PN: SINGLE CODE> <PN: ASK ALL RESPONDENTS>**

| Not at all severe |  |  |  |  |  | Extremely severe |
| --- | --- | --- | --- | --- | --- | --- |
| 1 | 2 | 3 | 4 | 5 | 6 | 7 |

1. Which of following are you currently experiencing during this **<PN: INSERT PHASE>** because of your depression?

**<PN: MULTI CODE> <PN: ASK ALL RESPONDENTS>**

| Withdrawal from social life | 1 |
| --- | --- |
| Relationship breakdown | 2 |
| Loss of employment | 3 |
| Inability to find employment | 4 |
| Difficulty functioning effectively at work or in studies | 5 |
| Difficulty functioning in social life | 6 |
| Difficulty functioning at home / in family life | 7 |
| Lack of intimacy with partner **<PN: Always anchor in this position>** | 8 |
| None of these | 9 |
| Unsure / don’t know | 10 |

1. What treatment(s) are you currently being prescribed by your doctor during this phase to treat your depression?

**<PN: SINGLE CODE> <PN: ASK ALL RESPONDENTS> <PN: SHOW ONLY LOCAL PRODUCT NAMES>**

| Molecule | Brands |  |
| --- | --- | --- |
| Agomelatine | <insert per specific market list> | 1 |
| Bupropion |  | 2 |
| Citalopram |  | 3 |
| Duloxetine |  | 4 |
| Escitalopram |  | 5 |
| Fluoxetine |  | 6 |
| Mirtazapine |  | 7 |
| Paroxetine |  | 8 |
| Sertraline |  | 9 |
| Venlafaxine |  | 10 |
| Vortioxetine |  | 11 |
| Atypical antidepressants |  | 12 |
| Benzodiazepines |  | 13 |
| Other drug therapy |  | 14 |
| Non-drug therapy (e.g. CBT) |  | 15 |
| Unsure / don’t know |  | 16 |
| None of the above |  | 17 |

1. What are the symptoms that you most want your treatment to address during this **<PN: INSERT PHASE>**?

**<PN: MULTI CODE> <PN: ASK ALL RESPONDENTS – ONLY SHOW RESPONSES GIVEN AT Q15> <PN: ROTATE STATEMENT ORDER>**

| Feeling sad / Low | 1 |
| --- | --- |
| Lack of energy | 2 |
| Lack of interest in general | 3 |
| Unable to take pleasure in things | 4 |
| Low self esteem | 5 |
| Lacking in confidence | 6 |
| Feelings of guilt or worthlessness | 7 |
| Suicidal thoughts | 8 |
|  |  |
| Reduced activity | 9 |
| Difficulty sleeping | 10 |
| Feeling tired | 11 |
| Waking early in the morning | 12 |
| Feeling agitated / Restless | 13 |
| Weight loss | 14 |
| Loss of sex drive | 15 |
| Loss of appetite | 16 |
| Depression worst in the morning | 17 |
|  |  |
| Forgetfulness / difficulty remembering | 18 |
| Slowness of thinking | 19 |
| Slowness in physical movements | 20 |
| Difficulty concentrating | 21 |
| Difficulty to prioritise / make decisions | 22 |
| Difficulty in making plans | 23 |
|  |  |
| Other (please specify) **<PN: ANCHOR>** | 24 |
| None of these symptoms **<PN: ANCHOR>** | 25 |

1. Which ONE of the following best reflects your hopes for treatment during this **<PN: INSERT PHASE>** phase?

**<PN: SINGLE CODE> <PN: ASK ALL RESPONDENTS> <PN: ROTATE STATEMENTS>**

| To lift my mood | 1 |
| --- | --- |
| To reduce side effects I am experiencing | 2 |
| To help me return to a normal working life | 3 |
| To help me return to a normal family life | 4 |
| To help me return to a normal social life (e.g. socialising with my friends etc.) | 5 |

1. Which symptoms, if any, do you feel your current treatment does not adequately treat during this **<PN: INSERT PHASE>**?

**<PN: MULTI CODE> <PN: ASK ALL RESPONDENTS – ONLY SHOW RESPONSES GIVEN AT Q15> <PN: ROTATE STATEMENT ORDER>**

| Feeling sad / Low | 1 |
| --- | --- |
| Lack of energy | 2 |
| Lack of interest in general | 3 |
| Unable to take pleasure in things | 4 |
| Low self esteem | 5 |
| Lacking in confidence | 6 |
| Feelings of guilt or worthlessness | 7 |
| Suicidal thoughts | 8 |
|  |  |
| Reduced activity | 9 |
| Difficulty sleeping | 10 |
| Feeling tired | 11 |
| Waking early in the morning | 12 |
| Feeling agitated / Restless | 13 |
| Weight loss | 14 |
| Loss of sex drive | 15 |
| Loss of appetite | 16 |
| Depression worst in the morning | 17 |
|  |  |
| Forgetfulness / difficulty remembering | 18 |
| Slowness of thinking | 19 |
| Slowness in physical movements | 20 |
| Difficulty concentrating | 21 |
| Difficulty to prioritise / make decisions | 22 |
| Difficulty in making plans | 23 |
|  |  |
| Other (please specify) **<PN: ANCHOR>** | 24 |
| None of these symptoms **<PN: ANCHOR>** | 25 |

1. To what extent are you experiencing difficulties with the following aspects during this **<PN: INSERT PHASE>**?

**<PN: MUTLI CODE> <PN: ASK ALL RESPONDENTS> <PN: ROTATE STATEMENT GROUPS AND STATEMENTS WITHIN GROUPS>**

| No difficulty | Mild Difficulty | Moderate difficulty | Severe difficulty | Don’t know |
| --- | --- | --- | --- | --- |
| 1 | 2 | 3 | 4 | 5 |

| **AUTONOMY** |  |
| --- | --- |
| Taking responsibility for the household | 1 |
| Living on my own | 2 |
| Doing the shopping | 3 |
| Taking care of myself and my appearance | 4 |
| **OCCUPATIONAL FUNCTIONING** |  |
| Holding down a paid job | 5 |
| Accomplishing tasks as quickly as necessary | 6 |
| Working in the field in which I am educated | 7 |
| Maintaining the amount of money I earn in relation to my job position | 8 |
| Managing my expected work load | 9 |
| **COGNITIVE FUNCTIONING** |  |
| Ability to concentrate on a book, film | 10 |
| Ability to make mental calculations | 11 |
| Ability to solve a problem adequately | 12 |
| Ability to remember newly-learned names | 13 |
| Ability to learn new information | 14 |
| **FINANCIAL ISSUES** |  |
| Managing my money | 15 |
| Spending my money in a balanced way | 16 |
| **INTERPERSONAL RELATIONSHIPS** |  |
| Maintaining a friendship or friendships | 17 |
| Participating in social activities | 18 |
| Having good relationships with people close to me | 19 |
| Living together with my family | 20 |
| Having satisfactory sexual relationships | 21 |
| Being able to defend my interests | 22 |
| **LEISURE TIME** |  |
| Doing exercise or participating in sport | 23 |
| Having hobbies or personal interests | 24 |

1. Please briefly describe the impact that depression is having on your work life. This may include anything from the types of activities and tasks you do at work to your relationships with other colleagues during this **<PN: INSERT PHASE>**

**<PN: OPEN END> <PN: ASK ALL RESPONDENTS CODING 1-2 AT Q3>**

|  |
| --- |

1. Please briefly describe the impact that depression is having on your family/home life. This may include anything from the types of conversations you have at home to how you interact generally with your family during this **<PN: INSERT PHASE>**

**<PN: OPEN END> <PN: ASK ALL RESPONDENTS>**

|  |
| --- |

1. Please briefly describe the impact that depression is having on your social life. This may include anything from the types of things you go out and do with your friends to the types of conversations you have during this **<PN: INSERT PHASE>**

**<PN: OPEN END> <PN: ASK ALL RESPONDENTS>**

|  |
| --- |

1. To what degree did you discuss each of the following issues with the doctor at the last appointment during this **<PN: INSERT PHASE>? <PN: SINGLE CODE> <PN: ASK ALL RESPONDENTS>**

| Did not discuss | Discussed a little | Discussed a lot |
| --- | --- | --- |
| 1 | 2 | 3 |

| The treatment options / medications available | 1 |
| --- | --- |
| Impact of depression on my family/home life | 2 |
| Impact of depression on my social life (e.g. socialising with friends) | 3 |
| Impact of depression on my work life | 4 |
| Side effects of the medications I am currently taking | 5 |
| Side effects of new medications I might take | 6 |

1. Which of the following, if any, did **YOU**, rather than your doctor raise at the last appointment during this **<PN: INSERT PHASE>**? **<PN: MULTI CODE> <PN: ASK ALL RESPONDENTS>**

| The treatment options / medications available | 1 |
| --- | --- |
| Impact of depression on my family/home life | 2 |
| Impact of depression on my social life (e.g. socialising with friends) | 3 |
| Impact of depression on my work life | 4 |
| Side effects of the medications I am currently taking | 5 |
| Side effects of new medications I might take | 6 |
| None of these | 7 |

1. I would now like you think back to the most recent appointment you had with your doctor about depression. Which of the following symptoms did you discuss at this time during this **<PN: INSERT PHASE>?**

**<PN: MULTI CODE> <PN: ASK ALL RESPONDENTS – ONLY SHOW RESPONSES GIVEN AT Q15> <PN: ROTATE STATEMENT ORDER>**

| Feeling sad / Low | 1 |
| --- | --- |
| Lack of energy | 2 |
| Lack of interest in general | 3 |
| Unable to take pleasure in things | 4 |
| Low self esteem | 5 |
| Lacking in confidence | 6 |
| Feelings of guilt or worthlessness | 7 |
| Suicidal thoughts | 8 |
|  |  |
| Reduced activity | 9 |
| Difficulty sleeping | 10 |
| Feeling tired | 11 |
| Waking early in the morning | 12 |
| Feeling agitated / Restless | 13 |
| Weight loss | 14 |
| Loss of sex drive | 15 |
| Loss of appetite | 16 |
| Depression worst in the morning | 17 |
|  |  |
| Forgetfulness / difficulty remembering | 18 |
| Slowness of thinking | 19 |
| Slowness in physical movements | 20 |
| Difficulty concentrating | 21 |
| Difficulty to prioritise / make decisions | 22 |
| Difficulty in making plans | 23 |
|  |  |
| Other (please specify) **<PN: ANCHOR>** | 24 |
| None of these symptoms **<PN: ANCHOR>** | 25 |

1. What specifically did you say to your doctor about the following symptoms that you discussed **<PN: SHOW SYMPTOMS MENTIONED AT CODEs 18-23 Q26>**

**<PN: OPEN END>**

|  |
| --- |

| Unsure / don’t remember | 2 |
| --- | --- |

1. How **satisfied** **were you with the discussion** you had with the doctor at the last appointment during this **<PN: INSERT PHASE>**? **<PN: SINGLE CODE> <PN: ASK ALL RESPONDENTS>**

| Very dissatisfied |  |  |  |  |  | Very satisfied | Unsure / don’t know |
| --- | --- | --- | --- | --- | --- | --- | --- |
| 1 | 2 | 3 | 4 | 5 | 6 | 7 | 8 |

1. To what extent did you feel that you had an **influence on the decision that was made** at this last appointment during this **<PN: INSERT PHASE> to prescribe <INSERT CODE FROM Q18>**?

**<PN: SINGLE CODE> <PN: ASK ALL RESPONDENTS>**

| Very uninfluential |  |  |  |  |  | Very influential | Unsure /Not relevant |
| --- | --- | --- | --- | --- | --- | --- | --- |
| 1 | 2 | 3 | 4 | 5 | 6 | 7 | 8 |

1. How **satisfied did you feel about the decision** that was made at the last appointment during this **<PN: INSERT PHASE> to prescribe <INSERT CODE FROM Q18>**? **<PN: SINGLE CODE> <PN: ASK ALL RESPONDENTS>**

| Very dissatisfied |  |  |  |  |  | Very satisfied | Unsure / don’t know |
| --- | --- | --- | --- | --- | --- | --- | --- |
| 1 | 2 | 3 | 4 | 5 | 6 | 7 | 8 |

1. Please indicate the extent to which you agree or disagree with each of the following statements that reflect your thoughts on your doctor during **<PN: INSERT PHASE>**:

**<PN: SINGLE CODE> <PN: ASK ALL RESPONDENTS>**

| Disagree completely |  |  | Indifferent |  |  | Agree completely |
| --- | --- | --- | --- | --- | --- | --- |
| 1 | 2 | 3 | 4 | 5 | 6 | 7 |

| I find it easy to discuss depression with my doctor | 1 |
| --- | --- |
| I have a good relationship with my doctor | 2 |
| I need a medication that will help me perform better at work **<PN: DO NOT SHOW IF CODE 3-6 AT Q6>** | 3 |
| I need a medication that will help me to have a more normal social life | 4 |
| I need a medication that will help me to have a better home/family life | 5 |
| I suffer primarily from low mood | 6 |
| My doctor listens to me | 7 |
| Appointments with my doctor are often difficult and unproductive | 8 |
| I would like to play more of a role in appointments with my doctor | 9 |
| My doctor is interested in **ALL** of my symptoms | 10 |
| It is just as important to treat my attention and concentration symptoms as it is my low mood symptoms | 11 |
| My attention and concentration symptoms have not been controlled by medication quite as well as my mood symptoms | 12 |
| I have never discussed attention and concentration symptoms with my doctor | 13 |

***Thank and close***

Thank you very much for taking part in this survey. If you have been affected by any of the issues raised in the survey the following organisations may be able to provide help and advice:

<Links to relevant support websites to be added for each country>

**Product References (for programming):**

| Molecule | Brazil | Canada | Mexico | S.K | USA | France | Italy | Spain |
| --- | --- | --- | --- | --- | --- | --- | --- | --- |
| Agomelatine | <to be inserted> | - |  |  |  | Valdoxan | Thymanax; Valdoxan | Thymanax; Valdoxan |
| Bupropion |  | Budeprion; Bupropion; Wellbutrin |  |  |  | - | Elontril; Wellbutrin | Elontril |
| Citalopram |  | Celexa; Cipramil; Citalopram; Ran; Ran-Citalo; Talohexal |  |  |  | Citalopram; Seropram | Citalopram; Citisint; Elopram; Felipram; Feliximir; Frimaind; Kaidor; Lampopram; Marpram; Percitale; Pramexyl; Return; Ricap; Seropram; Sintopram; Verisan; Vivipram | Calton; Citaolpram; Citalvir; Genprol; Prisdal; Relapaz; Seregra; Seropram |
| Duloxetine |  | Cymbalta |  |  |  | Cymbalta | Cymbalta; Xeristar | Cymbalta; Xeristar |
| Escitalopram |  | Cipralex |  |  |  | Seroplex | Cipralex; Entact | Cipralex; Escilan; Escimylan; Escitalopram; Esertia; Heipram |
| Fluoxetine |  | Auscap; Erocap; Fluohexal; Fluoxetine; FXT 20; FXT 40; Lovan; Prozac; Zactin |  |  |  | Fluoxetine; Prozac | Azur; Clexiclor; Cloriflox; Deprexen; Diesan; Flotina; Fluoxetine; Prozac; Xeredien | Adofen; Astrin; Fluoxetine; Lecimar; Luramon; Nodepe; Prozac; Reneuron |
| Mirtazapine |  | Avanza; Mirtazapine; Remeron |  |  |  | Mirtazapine; Norset | Mirtazapine; Remeron; Zatimar | Afloyan; Mirtamylan; Mirtazapine; Rexer; Vastat |
| Paroxetine |  | Aropax; Paroxetine; Paxil; Paxtine |  |  |  | Deroxat; Divarius;Paroxatine | Dapagut; Daparox; Dropaxin; Eutimil; Paroxetine; Serestill; Serupin; Seroxat; Stiliden | Arapaxel; Casbol; Daparox;; Frosinor; Motivan; Paroxteine; Seroxat; Xetin; Zuria |
| Sertraline |  | Sertraline; Zoloft |  |  |  | Sertraline; Zoloft | Serad; Sertraline; Tatig; Tralforin; Tralisen; Zoloft | Altisben; Aremis; Aserin; Besitran; Sertraline |
| Venlafaxine |  | Effexor; Venlafaxine; Venlafaxine SR |  |  |  | Efexor; Venlafaxine | Efexor; Faxine; Venlafaxine | Arafaxina; Conervin; Dislaven; Dobupal; Flaxen; Levest; Vandral; Venlabrain; Venlafaxine; Venlamylan; Zarelis |
| Vortioxetine |  |  |  |  |  | Brintellix |  |  |

**Quotas:**

| Country | Total | Acute | Post-acute | Remission |
| --- | --- | --- | --- | --- |
| Brazil | 250 | Minimum 50 | Minimum 50 | Minimum 50 |
| Canada | 250 | Minimum 50 | Minimum 50 | Minimum 50 |
| Mexico | 250 | Minimum 50 | Minimum 50 | Minimum 50 |
| South Korea | 250 | Minimum 50 | Minimum 50 | Minimum 50 |
| USA | 250 | Minimum 50 | Minimum 50 | Minimum 50 |
| France | 250 | Minimum 50 | Minimum 50 | Minimum 50 |
| Italy | 250 | Minimum 50 | Minimum 50 | Minimum 50 |
| Spain | 250 | Minimum 50 | Minimum 50 | Minimum 50 |

**Supplementary appendix C**

**HCP questionnaire**

**PR 0072 MDD HCP and Patient Journey Research: HCP Questionnaire**

**Section 1: Introduction, screening and current practice (5 minutes)**

Thank you for agreeing to take part in this research, this survey is conducted by BPR on behalf of a pharmaceutical company and will focus on the area of major depressive disorder (MDD) **<PN: In Mexico do not translate MDD, translate as ‘Depression’/ US and Canada ‘MDD’, EU markets ‘MDE’>**. The survey should take approximately 30 minutes to complete and all of your personal details will remain confidential and will not be divulged or passed onto the client or any other organisation.

Before we begin, we need to ask you a short series of questions to check your suitability for the survey.

**Adverse event reporting**

We are required to pass on to our client details of adverse events/product complaints pertaining to their products that are mentioned during the course of market research. Although what you say will, of course, be treated in confidence, should you raise during this survey an adverse event or product complaint in a specific patient, or group of patients, we will need to report this even if it has already been reported by you directly to the company or the regulatory authorities.

S1. Please confirm you are happy to continue on this basis?

**<PN: SINGLE CODE> <PN: ASK ALL RESPONDENTS>**

| Yes | 1 | **Continue** |
| --- | --- | --- |
| No | 2 | **Screen out** |

S2a. Are you currently employed by a pharmaceutical company or market research agency? This may include performing duties as a speaker, opinion leader of advisory board member

**<PN: SINGLE CODE> <PN: ASK ALL RESPONDENTS>**

| Yes | 1 | **Screen out** |
| --- | --- | --- |
| No | 2 | **Continue** |

**<PN: S2b- US ONLY QUESTION>**

S2b. Are you, or is a member of your immediate family, currently or within the past 6 months employed by or under contract with any of the following?

**<PN: MUTLI CODE> <PN: ASK ALL RESPONDENTS>**

| The FDA | 1 | **Screen out** |
| --- | --- | --- |
| The Federal Government or agencies of the Federal Government including VA hospitals | 2 | **Screen out** |
| A State Government or agencies of a State Government | 3 | **Screen out** |
| A marketing or market research department or company | 4 | **Screen out** |
| An advertising agency | 5 | **Screen out** |
| As a clinical investigator conducting clinical trials or providing consulting services for a depression drug | 6 | **Screen out** |
| A pharmaceutical company or healthcare manufacturer | 7 | **Screen out** |
| None of the above | 8 | **Continue** |

S3a. What is your primary medical specialty?

**<PN: SINGLE CODE> <PN: ASK ALL RESPONDENTS>**

| Primary care practitioner | 1a | **Continue (S. Korea: Screen out)** |
| --- | --- | --- |
| General Practice **<PN SHOW IN US ONLY>** | 1b | **Continue (class as PCP)** |
| Family Practice **<PN SHOW IN US ONLY>** | 1c | **Continue (class as PCP)** |
| Internal Medicine **<PN SHOW IN US ONLY>** | 1d | **Continue (class as PCP)** |
| Psychiatrist | 2 | **Continue** |
| Other | 3 | **Screen out** |

S3b. What proportion of of your time is spent in clinical practice performing direct patient care?

**<PN: NUMERIC> <PN: ASK ALL RESPONDENTS> <PN: THANK AND CLOSE IF LESS THAN 75%>**

|  |  | *%* |
| --- | --- | --- |

**<PN: S3B - US ONLY QUESTION – ONLY ASK IF CODING 1A-1D AT S3A>**

S3c . Do you have a board-certified subspecialty in Psychiatry? **<PN: SINGLE CODE>**

| Yes | 1 | **Continue to s3d.** |
| --- | --- | --- |
| No | 2 | **Class as PCP** |

**<PN: S3C- US ONLY QUESTION>**

S3d . Do you spend more than 25% of your time devoted to psychiatry? **<PN: SINGLE CODE>**

| Yes | 1 | **Class as Psychiatrist** |
| --- | --- | --- |
| No | 2 | **Class as PCP** |

**<PN: S3D - US ONLY QUESTION>**

S3d . Please select all the states in which you practice and /or are licensed below: **<PN: MULTI CODE>**

| Alabama | 1 | Montana | 27 |
| --- | --- | --- | --- |
| Alaska | 2 | Nebraska | 28 |
| Arizona | 3 | Nevada | 29 |
| Arkansas | 4 | New Hampshire | 30 |
| California | 5 | New Jersey | 31 |
| Colorado | 6 | New Mexico | 32 |
| Connecticut | 7 | New York | 33 |
| D.C. - District of Columbia **Screen out** | 8 | North Carolina | 34 |
| Delaware | 9 | North Dakota | 35 |
| Florida | 10 | Ohio | 36 |
| Georgia | 11 | Oklahoma | 37 |
| Hawaii | 12 | Oregon | 38 |
| Idaho | 13 | Pennsylvania | 39 |
| Illinois | 14 | Rhode Island | 40 |
| Indiana | 15 | South Carolina | 41 |
| Iowa | 16 | South Dakota | 42 |
| Kansas | 17 | Tennessee | 43 |
| Kentucky | 18 | Texas | 44 |
| Louisiana | 19 | Utah | 45 |
| Maine **Screen out** | 20 | Vermont **Screen out** | 46 |
| Maryland | 21 | Virginia | 47 |
| Massachusetts **Screen out** | 22 | Washington | 48 |
| Michigan | 23 | West Virginia **Screen out** | 49 |
| Minnesota **Screen out** | 24 | Wisconsin | 50 |
| Mississippi | 25 | Wyoming | 51 |
| Missouri | 26 |  |  |

S4. Do you work primarily in an office, a hospital or do you work in both an office and hospital?

**<PN: SINGLE CODE> <PN: ASK ALL RESPONDENTS>**

| Office only | 1 | **<PN: 50/50 QUOTA FOR HOSPITAL / OFFICE SPLIT -TO BE MONITORED CLOSELY>** |
| --- | --- | --- |
| Hospital / hospital clinic only | 2 |  |
| Both office and hospital | 3 |  |

S5. Are you personally responsible for prescribing antidepressant medication to treat major depressive disorder **<PN: Mexico MDD = ‘Depression’/ US and Can ‘MDD’, EU ‘MDE’>** **<PN: SINGLE CODE>** **<PN: ASK ALL RESPONDENTS>**

| Yes | 1 | **Continue** |
| --- | --- | --- |
| No | 2 | **Screen out** |

S6. Approximately how many patients diagnosed with **Major Depressive Disorder** **(MDD)** **<PN: Mexico MDD = ‘Depression’/ US and Can ‘MDD’, EU ‘MDE’>** do you personally manage and treat in a typical month?

**<PN: NUMERIC. RANGE 0-500> <PN: ASK ALL RESPONDENTS>**

|  |  |  |
| --- | --- | --- |

**<PN: FOR PCPS (CODING 1 AT S3) SCREEN OUT IF 14 OR LESS/PSYCHIATRISTS (CODING 2 AT S3) SCREEN OUT IF 39 OR LESS>**

S7. Approximately what proportion of your patients with MDD **<PN: Mexico MDD = ‘Depression’/ US and Can ‘MDD’, EU ‘MDE’>** do you prescribe anti-depressant medication to?

**<PN: NUMERIC. RANGE 0-100%> <PN: ASK ALL RESPONDENTS>**

|  |  |  |  | % |
| --- | --- | --- | --- | --- |

**<PN: SCREEN OUT IF 74% OR LOWER>**

**<PN: S8 - US ONLY QUESTION>**

S8. What percentage of the time do you spend treating patients in each of the following settings?

**<PN: NUMERIC. RANGE 0-100%> <PN: ASK ALL RESPONDENTS>**

|  | What percentage of the time do you spend treating patients in each of the following settings? | | |
| --- | --- | --- | --- |
| *Please enter a % for each row.* **<PN RANDOMIZE, MUST SUM TO 100%>** | | |  |
|  | Private or shared office |  |  |
|  | Clinic |  |  |
|  | Hospital |  |  |
|  | Nursing home | **Screen out if more than 10%** |  |
|  | Long-term care facility | **Screen out if more than 10%** |  |
|  | Other | **Screen out if more than 10%** |  |

**Patient Case Records**

As part of this research you will be asked questions regarding specific adult patients who have been diagnosed with **MDD <PN: Mexico MDD = ‘Depression’/ US and Can ‘MDD’, EU ‘MDE’>** and are receiving or have received **antidepressant treatment**. In order to complete these questions you will need to refer to **3 individual patient case records**. Each patient must be unique have been diagnosed with MDD **<PN: Mexico MDD = ‘Depression’/ US and Can ‘MDD’, EU ‘MDE’>** and you must be responsible for the management and treatment of this patient.

We would like each patient case record to represent a different patient type as listed below:

- A patient in the **ACUTE** phase of the condition. By **acute phase** we mean: a patient experiencing acute symptoms of major depressive disorder that require antidepressant treatment
- A patient in the **POST-ACUTE** phase of the condition. By **post-acute phase** we mean: post-acute phase where the patient is responding to antidepressant treatment with some symptom reduction but has not yet reached remission
- A patient in the **REMISSION** phase of the condition. By **remission phase** we mean: the patient feels better and experiences a significant reduction in symptoms compared to the acute or post-acute phase. Some residual symptoms may persist but are significantly fewer in number and severity compared to other phases

**<PN: THROUGHOUT QUESTIONNAIRE, WHEN HOVERING OVER A PHASE MAKE THE DEFINITION POP OUT>**

You will need access to these patient records to complete the survey. Please take time to ensure you have access to these patient records before proceeding with the survey. If you need time to collect these records you will have the ability to pause the survey and return later on.

S9. Can you please confirm that you will have access to 3 individual patient case records as listed above?

| Yes | 1 | **Continue** |
| --- | --- | --- |
| No | 2 | **Screen out** |

**Section 2: Introduction and current practice continued…. (qualified for full survey)**

1. Now, please think of the MDD **<PN: Mexico MDD = ‘Depression’/ US and Can ‘MDD’, EU ‘MDE’>** patients you see and treat in a typical month, approximately what proportion of these fall into each of the following categories?

**<PN: NUMERIC. MUST TOTAL 100%> <PN: ASK ALL RESPONDENTS>**

|  |  |  | % | Are currently experiencing an **ACUTE** phase | |
| --- | --- | --- | --- | --- | --- |
|  |  |  |  |  | |
|  |  |  | % | Are currently experiencing a **POST ACUTE** phase | |
|  |  |  |  |  | |
|  |  |  | % | Are currently experiencing a **REMISSION** phase |  |

- A patient in the **ACUTE** phase of the condition. By **acute phase** we mean: a patient experiencing acute symptoms of major depressive disorder that require antidepressant treatment
- A patient in the **POST ACUTE** phase of the condition. By **post acute phase** we mean: post-acute phase where the patient is responding to antidepressant treatment with some symptom reduction but has not yet reached remission
- A patient in the **REMISSION** phase of the condition. By **remission phase** we mean: the patient feels better and experiences a significant reduction in symptoms compared to the acute or post-acute phase. Some residual symptoms may persist but are significantly fewer in number and severity compared to other phases

**Section 3/4/5: Patient Case Records**

In this section we would like to ask you some questions about 3 of your patients with MDD **<PN: Mexico MDD = ‘Depression’/ US and Can ‘MDD’, EU ‘MDE’>**. These patients should be aged 18 years or more **<PN: aged 25 to 70 in the US>**, diagnosed with MDD **<PN: Mexico MDD = ‘Depression’/ US and Can ‘MDD’, EU ‘MDE’>** and be receiving or have received antidepressant medication for major depressive disorder. You must be responsible for the management and treatment of this patient and each patient must be unique.

**<PN: PLEASE ALLOW CAPABILITY TO LOG OFF/PAUSE SURVEY TO RETRIEVE PATIENT CASE RECORDS IF NEED BE>**

**Section 3: Patient Case Record #1 ‘Acute’ (8 minutes)**

**<PN: PLEASE ROTATE THE ORDER IN WHICH SECTIONS 3,4 AND 5 ARE SHOWN TO THE RESPONDENT, SO THAT EACH DIFFERENT TYPE OF CASE RECORD IS ROTATED>**

**<PN: PLEASE PROGRAMME AT THE TOP OF EACH PAGE A NOTE TO REMIND THE RESPONDENT ABOUT WHICH CASE RECORD IS BEING DISCUSSED – ACUTE / POST ACUTE / REMISSION>**

To begin with we would like you to focus on **<PN: ROTATE PCR 1/2/3 ORDER>**

Next we would like you to focus on **<PN: ROTATE PCR 1/2/3 ORDER>**

…the last MDD **<PN: Mexico MDD = ‘Depression’/ US and Can ‘MDD’, EU ‘MDE’>** patient you consulted with who was in the **ACUTE** phase of the condition during this consultation. By **Acute phase** we mean: a patient experiencing acute symptoms of major depressive disorder that require antidepressant treatment

**Background information about the patient**

1. How long ago did this consultation with the patient take place *(please enter as appropriate)*?

**<PN: SINGLE CODE> <PN: ASK ALL RESPONDENTS>**

|  |  | Weeks |  |  | Months |  |  | Years |
| --- | --- | --- | --- | --- | --- | --- | --- | --- |

1. How old is this patient?

**<PN: NUMERIC. RANGE 18-90/25-70 in the US > <PN: ASK ALL RESPONDENTS>**

|  |  |
| --- | --- |

1. Indicate the patient’s gender

**<PN: SINGLE CODE> <PN: ASK ALL RESPONDENTS>**

| Male | 1 |
| --- | --- |
| Female | 2 |

1. Which of the following best describes the patient’s highest level of education? <**PN: SINGLE CODE> <PN: ASK ALL RESPONDENTS>**

| Post-graduate | 1 |
| --- | --- |
| Undergraduate | 2 |
| High School Graduate | 3 |
| Vocational Qualification | 4 |
| No qualifications | 5 |
| Other | 6 |
| Don’t know | 7 |

1. Which of the following best describes the patient’s current working status? <**PN: SINGLE CODE> <PN: ASK ALL RESPONDENTS>**

| Full-time (30 or more hours per week) | 1 | **Go to Q7** |
| --- | --- | --- |
| Part-time | 2 | **Go to Q7** |
| Retired | 3 | **Go to Q8** |
| Homemaker/ Stay-at-home parent | 4 | **Go to Q8** |
| Student | 5 | **Go to Q8** |
| Unemployed | 6 | **Go to Q8** |

1. Which of the following occupational groups best reflects the type of job the patient does

<**PN: SINGLE CODE> <PN: ASK ALL RESPONDENTS>**

| **Semi or unskilled manual work** (e.g. Manual workers, apprentices to be skilled trades, Shop assistants etc.) | 1 |
| --- | --- |
| **Skilled manual worker** (e.g. Plumber, Lorry driver, Mechanic etc.) | 2 |
| **Supervisory or clerical** junior managerial/administrative (e.g. Office worker, Student Doctor, Foreman etc.) | 3 |
| **Intermediate managerial/ professional/ administrative** (e.g. Newly qualified doctor, Solicitor, Middle manager in large organisation) | 4 |
| **Higher managerial/ professional/ administrative** (e.g. Established doctor, Solicitor, Board Director in a large organisation, top level civil servant) | 5 |
| **Student** | 6 |
| **Casual worker** (not in permanent employment) | 7 |
| **Homemaker/ stay at home parent** | 8 |
| **Retired** | 9 |
| **Unemployed or not working due to long-term sickness** | 10 |
| **Other** | 11 |

1. Approximately how long ago was this patient first diagnosed with MDD **<PN: Mexico MDD = ‘Depression’/ US and Can ‘MDD’, EU ‘MDE’>**  *(please enter as appropriate)*?

**<PN: NUMERIC. RANGE 0-90> <PN: ASK ALL RESPONDENTS>**

|  |  | Years |  |  |  | Months |  |  | Don’t know |
| --- | --- | --- | --- | --- | --- | --- | --- | --- | --- |

1. For approximately how long has the patient been experiencing this ACUTE phase of MDD**<PN: Mexico MDD = ‘Depression’/ US and Can ‘MDD’, EU ‘MDE’>**  *(please enter as appropriate)*?

**<PN: NUMERIC. RANGE 0-90> <PN: ASK ALL RESPONDENTS>**

|  |  | Weeks |  |  | Months |  |  | Years |
| --- | --- | --- | --- | --- | --- | --- | --- | --- |

1. Did you personally diagnose this ACUTE phase?

**<PN: SINGLE CODE> <PN: ASK ALL RESPONDENTS>**

| Yes | 1 |
| --- | --- |
| No | 2 |

1. What type of doctor diagnosed this patient at the ACUTE phase?

**<PN: SINGLE CODE> <PN: ASK ONLY RESPONDENTS WHO CODE 2 AT Q10>**

| Primary care physician | 1 |
| --- | --- |
| Psychiatrist | 2 |
| Neurologist | 3 |
| Geriatrician | 4 |
| Internal Medicine | 5 |
| Another specialist | 6 |
| Other | 7 |
| Don’t know | 8 |

1. Did this patient present directly to you for this ACUTE phase or were they referred to you from another physician?

**<PN: SINGLE CODE> <PN: ASK ALL RESPONDENTS>**

| Presented directly to me | 1 |
| --- | --- |
| Were referred by another physician | 2 |

1. What type of physician referred the patient on to you for this ACUTE phase?

**<PN: SINGLE CODE> <PN: ASK ALL RESPONDENTS WHO CODE 2 AT Q12>**

| Primary care physician | 1 |
| --- | --- |
| Psychiatrist | 2 |
| Neurologist | 3 |
| Psychologist | 4 |
| Another specialist | 5 |
| Geriatrician | 6 |
| Internal Medicine | 7 |
| Other | 8 |
| Don’t know | 9 |

1. For approximately how long have you been managing this patient?

**<PN: SINGLE CODE> <PN: ASK ALL RESPONDENTS>**

|  |  | Weeks |  |  | Months |  |  | Years |
| --- | --- | --- | --- | --- | --- | --- | --- | --- |

1. Is this the first ACUTE phase of MDD **<PN: Mexico MDD = ‘Depression’/ US and Can ‘MDD’, EU ‘MDE’>** that the patient has suffered or have they had previous ACUTE phases?

**<PN: SINGLE CODE> <PN: ASK ALL RESPONDENTS>**

| First time | 1 |
| --- | --- |
| Previous ACUTE phases | 2 |

1. Approximately how many previous ACUTE phases (excluding the current acute phase) have they had prior to the most recent ACUTE phase?

**<PN: SINGLE CODE> <PN: ASK ALL RESPONDENTS WHO CODE 2 AT Q15>**

|  |  | ACUTE phases |
| --- | --- | --- |

| Don’t know | 1 |
| --- | --- |

1. Has the patient’s working status changed because of this ACUTE phase of depression?

<**PN: MUTLI CODE> <PN: ASK ALL RESPONDENTS>**

| **Yes** – they have had to **reduce their working hours** | 1 |
| --- | --- |
| **Yes** – they have had to **take a** **less demanding job** | 2 |
| **Yes** – They have had to **take sick leave** | 3 |
| **Yes** – They have had to **stop working** | 4 |
| **No** – their working status **has not changed** | 5 |
| Don’t know | 6 |

**Information about ACUTE phase**

Please remember that with this patient we are focusing on the **ACUTE** phase of the condition. By **Acute phase** we mean: a patient experiencing acute symptoms of major depressive disorder that require antidepressant treatment

1. Which of the following symptoms did this patient experience during this **ACUTE** phase of depression?

**<PN: MULTI CODE> <PN: ASK ALL RESPONDENTS> <PN: ROTATE ORDER IN WHICH ‘GROUPS’ OF SYMPTOMS SHOWN AND ORDER OF INDIVIDUAL SYMPTOMS WITHIN GROUPS>**

| Feeling sad / Low | 1 |
| --- | --- |
| Lack of energy | 2 |
| Lack of interest in general | 3 |
| Unable to take pleasure in things | 4 |
| Low self esteem | 5 |
| Lacking in confidence | 6 |
| Feelings of guilt or worthlessness | 7 |
| Suicidal thoughts | 8 |
|  |  |
| Reduced activity | 9 |
| Difficulty sleeping | 10 |
| Feeling tired | 11 |
| Waking early in the morning | 12 |
| Feeling agitated / Restless | 13 |
| Weight loss | 14 |
| Loss of libido | 15 |
| Loss of appetite | 16 |
| Depression worst in the morning | 17 |
|  |  |
| Forgetfulness / difficulty remembering | 18 |
| Slowness of thinking | 19 |
| Slowness in physical movements | 20 |
| Difficulty concentrating | 21 |
| Difficulty to prioritise / make decisions | 22 |
| Difficulty in making plans | 23 |
|  |  |
| Other (please specify) **<PN: ANCHOR>** | 24 |

1. Overall, how severe would you rate the symptoms that the patient is experiencing in this ACUTE phase?

**<PN: SINGLE CODE> <PN: ASK ALL RESPONDENTS>**

| Not at all severe |  |  |  |  |  | Extremely severe |
| --- | --- | --- | --- | --- | --- | --- |
| 1 | 2 | 3 | 4 | 5 | 6 | 7 |

1. Which of the following has the patient experienced because of this ACUTE phase of depression?

**<PN: MULTI CODE> <PN: ASK ALL RESPONDENTS> <PN: ROTATE STATEMENTS>**

| Withdrawal from social life | 1 |
| --- | --- |
| Relationship breakdown | 2 |
| Loss of employment | 3 |
| Inability to find employment | 4 |
| Difficulty functioning effectively at work or in studies | 5 |
| Difficulty functioning in social life | 6 |
| Difficulty functioning at home / in family life | 7 |
| Lack of intimacy with partner **<PN: Always anchor in this position>** | 8 |
| None of these | 9 |
| Unsure / don’t know | 10 |

1. What treatment(s) did you prescribe to the patient during this ACUTE phase of depression?

**<PN: MULTI CODE> <PN: ASK ALL RESPONDENTS> <PN: SHOW ONLY LOCAL PRODUCT NAMES>**

| Molecule | Brands |  |
| --- | --- | --- |
| Agomelatine | <insert per specific market list> | 1 |
| Bupropion |  | 2 |
| Citalopram |  | 3 |
| Duloxetine |  | 4 |
| Escitalopram |  | 5 |
| Fluoxetine |  | 6 |
| Mirtazapine |  | 7 |
| Paroxetine |  | 8 |
| Sertraline |  | 9 |
| Venlafaxine |  | 10 |
| Vortioxetine |  | 11 |
| Atypical antidepressants |  | 12 |
| Benzodiazepines |  | 13 |
| Other drug therapy |  | 14 |
| Non-drug therapy (e.g. CBT) |  | 15 |
| No treatment or therapy prescribed |  | 16 |

1. Which of the following best describes the treatment decision made during this ACUTE phase to prescribe **<PN: INSERT PRODUCT(S) SELECTED FROM Q21>**?

**<PN: MULTI CODE> <PN: ASK ALL RESPONDENTS WHO CODE 1-14 AT Q21>**

| The patient is newly diagnosed and was prescribed pharmacological treatment for the first time | 1 |
| --- | --- |
| A repeat prescription was given as the patient has been responding well to this treatment | 2 |
| The dosage/formulation of the treatment was changes to achieve a better response | 3 |
| Another antidepressant was added to their existing treatment to achieve a better response | 4 |
| A different medication was added to their existing treatment – such as an anti-psychotic or mood stabilizer to achieve a better response | 5 |
| The patient was switched from one pharmacological treatment to another | 6 |
| Pharmacological treatment was re-started after the patient went for a period without taking medication | 7 |

1. What are the symptoms that you most want to address with the treatment prescribed during this ACUTE phase?

**<PN: MULTI CODE> <PN: ASK ALL RESPONDENTS – ONLY SHOW RESPONSES GIVEN AT Q18> <PN: ROTATE ORDER IN WHICH ‘GROUPS’ OF SYMPTOMS SHOWN AND ORDER OF INDIVIDUAL SYMPTOMS WITHIN GROUPS>**

| Feeling sad / Low | 1 |
| --- | --- |
| Lack of energy | 2 |
| Lack of interest in general | 3 |
| Unable to take pleasure in things | 4 |
| Low self esteem | 5 |
| Lacking in confidence | 6 |
| Feelings of guilt or worthlessness | 7 |
| Suicidal thoughts | 8 |
|  |  |
| Reduced activity | 9 |
| Difficulty sleeping | 10 |
| Feeling tired | 11 |
| Waking early in the morning | 12 |
| Feeling agitated / Restless | 13 |
| Weight loss | 14 |
| Loss of libido | 15 |
| Loss of appetite | 16 |
| Depression worst in the morning | 17 |
|  |  |
| Forgetfulness / difficulty remembering | 18 |
| Slowness of thinking | 19 |
| Slowness in physical movements | 20 |
| Difficulty concentrating | 21 |
| Difficulty to prioritise / make decisions | 22 |
| Difficulty in making plans | 23 |
|  |  |
| Other (please specify) **<PN: ANCHOR>** | 24 |

1. Which ONE of the following best reflects your primary treatment goal during this ACUTE phase?

**<PN: SINGLE CODE> <PN: ASK ALL RESPONDENTS> <PN: ROTATE STATEMENTS>**

| To elevate the patient’s mood | 1 |
| --- | --- |
| To reduce side effects | 2 |
| To return the patient to work | 3 |
| To return the patient to normal family life | 4 |
| To return the patient to normal social life | 5 |

1. Which of the following symptoms that this patient has experienced during this ACUTE phase of depression, if any, can you NOT adequately treat?

**<PN: MULTI CODE> <PN: ROTATE STATEMENT ORDER - ONLY SHOW RESPONSES GIVEN AT Q18> <PN: ROTATE ORDER IN WHICH ‘GROUPS’ OF SYMPTOMS SHOWN AND ORDER OF INDIVIDUAL SYMPTOMS WITHIN GROUPS>**

| No symptoms – I can adequately treat them all | 25 |
| --- | --- |
|  |  |
| Feeling sad / Low | 1 |
| Lack of energy | 2 |
| Lack of interest in general | 3 |
| Unable to take pleasure in things | 4 |
| Low self esteem | 5 |
| Lacking in confidence | 6 |
| Feelings of guilt or worthlessness | 7 |
| Suicidal thoughts | 8 |
|  |  |
| Reduced activity | 9 |
| Difficulty sleeping | 10 |
| Feeling tired | 11 |
| Waking early in the morning | 12 |
| Feeling agitated / Restless | 13 |
| Weight loss | 14 |
| Loss of libido | 15 |
| Loss of appetite | 16 |
| Depression worst in the morning | 17 |
|  |  |
| Forgetfulness / difficulty remembering | 18 |
| Slowness of thinking | 19 |
| Slowness in physical movements | 20 |
| Difficulty concentrating | 21 |
| Difficulty to prioritise / make decisions | 22 |
| Difficulty in making plans | 23 |
|  |  |
| Other (please specify) **<PN: ANCHOR>** | 24 |

1. As far as you are aware, which of the following symptoms does the patient most want treated during this ACUTE phase?

**<PN: MULTI CODE> <PN: ASK ALL RESPONDENT S- ONLY SHOW RESPONSES GIVEN AT Q18> <PN: ROTATE ORDER IN WHICH ‘GROUPS’ OF SYMPTOMS SHOWN AND ORDER OF INDIVIDUAL SYMPTOMS WITHIN GROUPS>**

| Feeling sad / Low | 1 |
| --- | --- |
| Lack of energy | 2 |
| Lack of interest in general | 3 |
| Unable to take pleasure in things | 4 |
| Low self esteem | 5 |
| Lacking in confidence | 6 |
| Feelings of guilt or worthlessness | 7 |
| Suicidal thoughts | 8 |
|  |  |
| Reduced activity | 9 |
| Difficulty sleeping | 10 |
| Feeling tired | 11 |
| Waking early in the morning | 12 |
| Feeling agitated / Restless | 13 |
| Weight loss | 14 |
| Loss of libido | 15 |
| Loss of appetite | 16 |
| Depression worst in the morning | 17 |
|  |  |
| Forgetfulness / difficulty remembering | 18 |
| Slowness of thinking | 19 |
| Slowness in physical movements | 20 |
| Difficulty concentrating | 21 |
| Difficulty to prioritise / make decisions | 22 |
| Difficulty in making plans | 23 |
|  |  |
| Other (please specify) **<PN: ANCHOR>** | 24 |

1. In your opinion, to what extent is the patient experiencing difficulties with the following aspects during the acute phase:

**<PN: MUTLI CODE> <PN: ASK ALL RESPONDENTS> <PN: ROTATE STATEMENT GROUPS AND STATEMENTS WITHIN GROUPS>**

| No difficulty | Mild Difficulty | Moderate difficulty | Severe difficulty | Don’t know |
| --- | --- | --- | --- | --- |
| 1 | 2 | 3 | 4 | 5 |

| **AUTONOMY** |  |
| --- | --- |
| Taking responsibility for his/her household | 1 |
| Living on his/her own | 2 |
| Doing his/her shopping | 3 |
| Taking care of themselves (physical aspects, hygiene) | 4 |
| **OCCUPATIONAL FUNCTIONING** |  |
| Holding down a paid job | 5 |
| Accomplishing tasks as quickly as necessary | 6 |
| Working in the field in which he/she were educated | 7 |
| Maintaining the amount of money he/she earns in relation to their job position | 8 |
| Managing his/her expected work load | 9 |
| **COGNITIVE FUNCTIONING** |  |
| Ability to concentrate on a book, film | 10 |
| Ability to make mental calculations | 11 |
| Ability to solve a problem adequately | 12 |
| Ability to remember newly-learned names | 13 |
| Ability to learn new information | 14 |
| **FINANCIAL ISSUES** |  |
| Managing his/her own money | 15 |
| Spending his/her money in a balanced way | 16 |
| **INTERPERSONAL RELATIONSHIPS** |  |
| Maintaining a friendship or friendships | 17 |
| Participating in social activities | 18 |
| Having good relationships with people close to him/her | 19 |
| Living together with his/her family | 20 |
| Having satisfactory sexual relationships | 21 |
| Being able to defend his/her interests | 22 |
| **LEISURE TIME** |  |
| Doing exercise or participating in sport | 23 |
| Having hobbies or personal interests | 24 |

1. To what degree did you discuss each of the following issues with the patient during this ACUTE phase?

**<PN: SINGLE CODE> <PN: ASK ALL RESPONDENTS>**

| Did not discuss | Discussed a little | Discussed a lot |
| --- | --- | --- |
| 1 | 2 | 3 |

| Treatment options | 1 |
| --- | --- |
| Impact of depression on family/home life | 2 |
| Impact of depression on social life (e.g. socialising with friends) | 3 |
| Impact of depression on work life | 4 |
| Side effects of existing treatments | 5 |
| Sid effects of new treatment options | 6 |

1. Which of the following topics, if any, did the patient raise with you at any point during this ACUTE phase

**<PN: MULTI CODE> <PN: ASK ALL RESPONDENTS>**

| Treatment options | 1 |
| --- | --- |
| Impact of depression on family/home life | 2 |
| Impact of depression on social life (e.g. socialising with friends) | 3 |
| Impact of depression on work life | 4 |
| Side effects of existing treatments | 5 |
| Sid effects of new treatment options | 6 |
| None of these | 7 |

1. How did the patient react to the decision to **<PN: INSERT TREATMENT DECISION FROM Q22>** that was made during this ACUTE phase?

**<PN: SINGLE CODE> <PN: ASK ALL RESPONDENTS>**

| Very negatively |  |  |  |  |  | Very positively |
| --- | --- | --- | --- | --- | --- | --- |
| 1 | 2 | 3 | 4 | 5 | 6 | 7 |

1. Please indicated the extent to which the patient influenced your decision to **<PN: INSERT TREATMENT DECISION FROM Q22>**?

**<PN: SINGLE CODE> <PN: ASK ALL RESPONDENTS>**

| Not at all influential |  |  |  |  |  | Very influential |
| --- | --- | --- | --- | --- | --- | --- |
| 1 | 2 | 3 | 4 | 5 | 6 | 7 |

1. Please review each of the following statements about your management of the patient and indicate the degree to which you agree or disagree with each:

**<PN: SINGLE CODE> <PN: ASK ALL RESPONDENTS>**

| Disagree completely |  |  | Indifferent |  |  | Agree completely |
| --- | --- | --- | --- | --- | --- | --- |
| 1 | 2 | 3 | 4 | 5 | 6 | 7 |

| This patient is good at verbalising the symptoms they experience | 1 |
| --- | --- |
| I enjoy managing and treating this patient | 2 |
| This patient needs a treatment that will help them function better at work **<PN: DO NOT SHOW IF CODE 3-6 AT Q6>** | 3 |
| This patient needs a treatment that will help them function better in their social life (e.g. socialising with friends) | 4 |
| This patient needs a treatment that will help them function better at home / in family life | 5 |
| I find this patient extremely challenging to manage and treat | 6 |
| This patient suffers primarily from mood symptoms | 7 |
| Consultations with this patient are often difficult and unproductive | 8 |
| I wish this patient would play more of an active role in our consultations | 9 |
| It is just as important to treat this patient’s cognitive symptoms as it is their mood symptoms | 10 |
| This patient’s cognitive symptoms have not been controlled by medication quite as well as their mood symptoms have been controlled | 11 |
| This patient has never discussed cognitive symptoms with me | 12 |

**Section 4: Patient Case Record #2 ‘Post-acute’ (8 minutes)**

**<PN: PLEASE ROTATE THE ORDER IN WHICH SECTIONS 3,4 AND 5 ARE SHOWN TO THE RESPONDENT, SO THAT EACH DIFFERENT TYPE OF CASE RECORD IS ROTATED>**

**<PN: PLEASE PROGRAMME AT THE TOP OF EACH PAGE A NOTE TO REMIND THE RESPONDENT ABOUT WHICH CASE RECORD IS BEING DISCUSSED – ACUTE / POST-ACUTE / REMISSION>**

To begin with we would like you to focus on **<PN: ROTATE PCR 1/2/3 ORDER>**

Next we would like you to focus on **<PN: ROTATE PCR 1/2/3 ORDER>**

**<PN: DISPLAY DEFINITION>**

…an MDD **<PN: Mexico MDD = ‘Depression’/ US and Can ‘MDD’, EU ‘MDE’>** patient who is in the **POST-ACUTE** phase of the condition. By **post-acute phase** we mean: post-acute phase where the patient is responding to antidepressant treatment with some symptom reduction but has not yet reached remission

**Background information about the patient**

1. How long ago did this consultation with the patient take place *(please enter as appropriate)*?

**<PN: SINGLE CODE> <PN: ASK ALL RESPONDENTS>**

|  |  | Weeks |  |  | Months |  |  | Years |
| --- | --- | --- | --- | --- | --- | --- | --- | --- |

1. How old is this patient?

**<PN: NUMERIC. RANGE 18-90/25-70 in the US > <PN: ASK ALL RESPONDENTS>**

|  |  |
| --- | --- |

1. Indicate the patient’s gender

**<PN: SINGLE CODE> <PN: ASK ALL RESPONDENTS>**

| Male | 1 |
| --- | --- |
| Female | 2 |

1. Which of the following best describes the patient’s highest level of education? <**PN: SINGLE CODE> <PN: ASK ALL RESPONDENTS>**

| Post-graduate | 1 |
| --- | --- |
| Undergraduate | 2 |
| High School Graduate | 3 |
| Vocational Qualification | 4 |
| No Qualifications | 5 |
| Other | 6 |
| Don’t know | 7 |

1. Which of the following best describes the patient’s current working status? **<PN: SINGLE CODE> <PN: ASK ALL RESPONDENTS>**

| Full-time (30 or more hours per week) | 1 | **Go to Q38** |
| --- | --- | --- |
| Part-time | 2 | **Go to Q38** |
| Retired | 3 | **Go to Q39** |
| Homemaker/ Stay-at-home parent | 4 | **Go to Q39** |
| Student | 5 | **Go to Q39** |
| Unemployed | 6 | **Go to Q39** |

1. Which of the following occupational groups best reflects the type of job the patient does?

**<PN: SINGLE CODE> <PN: ASK ALL RESPONDENTS>**

| **Semi or unskilled manual work** (e.g. Manual workers, apprentices to be skilled trades, Shop assistants etc.) | 1 |
| --- | --- |
| **Skilled manual worker** (e.g. Plumber, Lorry driver, Mechanic etc.) | 2 |
| **Supervisory or clerical** junior managerial/administrative (e.g. Office worker, Student Doctor, Foreman etc.) | 3 |
| **Intermediate managerial/ professional/ administrative** (e.g. Newly qualified doctor, Solicitor, Middle manager in large organisation) | 4 |
| **Higher managerial/ professional/ administrative** (e.g. Established doctor, Solicitor, Board Director in a large organisation, top level civil servant) | 5 |
| **Student** | 6 |
| **Casual worker** (not in permanent employment) | 7 |
| **Homemaker/ stay at home parent** | 8 |
| **Retired** | 9 |
| **Unemployed or not working due to long-term sickness** | 10 |
| **Other** | 11 |

1. Approximately how long ago was this patient diagnosed with MDD **<PN: Mexico MDD = ‘Depression’/ US and Can ‘MDD’, EU ‘MDE’>**  *(please enter as appropriate)*?

**<PN: NUMERIC. RANGE 0-90> <PN: ASK ALL RESPONDENTS>**

|  |  | Years |  |  |  | Months |  |  | Don’t know |
| --- | --- | --- | --- | --- | --- | --- | --- | --- | --- |

1. For approximately how long has this patient responded to treatment during this **post-acute** phase of MDD *(please enter as appropriate)*?

**<PN: NUMERIC> <PN: ASK ALL RESPONDENTS>**

|  |  | Weeks |  |  |  | Months |  |  | Years |
| --- | --- | --- | --- | --- | --- | --- | --- | --- | --- |

1. Did you personally initiate the treatment that this patient is currently responding too?

**<PN: SINGLE CODE> <PN: ASK ALL RESPONDENTS>**

| Yes | 1 |
| --- | --- |
| No | 2 |

1. What type of doctor initiated the treatment that this patient is responding too?

**<PN: SINGLE CODE> <PN: ASK ALL RESPONDENTS CODING 2 AT Q42>**

| Primary care physician | 1 |
| --- | --- |
| Psychiatrist | 2 |
| Neurologist | 3 |
| Geriatrician | 4 |
| Internal Medicine | 5 |
| Another specialist | 6 |
| Other | 7 |
| Don’t know | 8 |

1. Did this patient present directly to you for thus POST-ACUTE phase or were they referred to you from another physician?

**<PN: SINGLE CODE> <PN: ASK ALL RESPONDENTS>**

| Presented directly to me | 1 |
| --- | --- |
| Were referred by another physician | 2 |

1. What type of physician referred the patient on to you for this POST-ACUTE phase?

**<PN: SINGLE CODE> <PN: ASK ALL RESPONDENTS WHO CODE 2 AT Q43>**

| Primary care physician | 1 |
| --- | --- |
| Psychiatrist | 2 |
| Neurologist | 3 |
| Psychologist | 4 |
| Another specialist | 5 |
| Other | 6 |
| Don’t know | 7 |

1. For approximately how long have you been managing this patient?

**<PN: SINGLE CODE> <PN: ASK ALL RESPONDENTS>**

|  |  | Weeks |  |  | Months |  |  | Years |
| --- | --- | --- | --- | --- | --- | --- | --- | --- |

1. Is this the first POST-ACUTE phase of MDD **<PN: Mexico MDD = ‘Depression’/ US and Can ‘MDD’, EU ‘MDE’>** that the patient has had or have they had previous POST-ACUTE phases?

**<PN: SINGLE CODE> <PN: ASK ALL RESPONDENTS>**

| First time |  | 1 | Go to Q48 |
| --- | --- | --- | --- |
| Previous POST-ACUTE phases |  | 2 | Go to Q47 |

1. Approximately how many previous POST-ACUTE PHASES (excluding the current POST-ACUTE phase) have they had prior to the most recent POST-ACUTE phase?

**<PN: SINGLE CODE> <PN: ASK ALL RESPONDENTS>**

|  |  | POST-ACUTE phases |
| --- | --- | --- |

| Don’t know | 1 |
| --- | --- |

1. Has the patient’s working status changed because of this POST-ACUTE phase of depression?

<**PN: MUTLI CODE> <PN: ASK ALL RESPONDENTS>**

| **Yes** – they have had to **reduce their working hours** | 1 |
| --- | --- |
| **Yes** – they have had to **take a** **less demanding job** | 2 |
| **Yes** – They have had to **take sick leave** | 3 |
| **Yes** – They have had to **stop working** | 4 |
| **No** – their working status **has not changed** | 5 |
| Don’t know | 6 |

**Information about POST-ACUTE phase**

Please remember that with this patient we are focusing on the **POST-ACUTE** phase of the condition. By post-acute phase where the patient is responding to antidepressant treatment with some symptom reduction but has not yet reached remission

1. Which of the following symptoms did this patient experience during this **POST-ACUTE** phase of depression?

**<PN: MULTI CODE> <PN: ASK ALL RESPONDENTS> <PN: ROTATE ORDER IN WHICH ‘GROUPS’ OF SYMPTOMS SHOWN AND ORDER OF INDIVIDUAL SYMPTOMS WITHIN GROUPS>**

| Feeling sad / Low | 1 |
| --- | --- |
| Lack of energy | 2 |
| Lack of interest in general | 3 |
| Unable to take pleasure in things | 4 |
| Low self esteem | 5 |
| Lacking in confidence | 6 |
| Feelings of guilt or worthlessness | 7 |
| Suicidal thoughts | 8 |
|  |  |
| Reduced activity | 9 |
| Difficulty sleeping | 10 |
| Feeling tired | 11 |
| Waking early in the morning | 12 |
| Feeling agitated / Restless | 13 |
| Weight loss | 14 |
| Loss of libido | 15 |
| Loss of appetite | 16 |
| Depression worst in the morning | 17 |
|  |  |
| Forgetfulness / difficulty remembering | 18 |
| Slowness of thinking | 19 |
| Slowness in physical movements | 20 |
| Difficulty concentrating | 21 |
| Difficulty to prioritise / make decisions | 22 |
| Difficulty in making plans | 23 |
|  |  |
| Other (please specify) **<PN: ANCHOR>** | 24 |
| No symptoms **<PN: ANCHOR>** | 25 |

1. Overall, how severe would you rate the symptoms that the patient is experiencing in this POST-ACUTE phase?

**<PN: SINGLE CODE> <PN: ASK ALL RESPONDENTS WHO CODE 1-24 at Q49>**

| Not at all severe |  |  |  |  |  | Extremely severe |
| --- | --- | --- | --- | --- | --- | --- |
| 1 | 2 | 3 | 4 | 5 | 6 | 7 |

1. Which of the following has the patient experienced during this POST-ACUTE phase of depression?

**<PN: MULTI CODE> <PN: ASK ALL RESPONDENTS>**

| Withdrawal from social life | 1 |
| --- | --- |
| Relationship breakdown | 2 |
| Loss of employment | 3 |
| Inability to find employment | 4 |
| Difficulty functioning effectively at work or in studies | 5 |
| Difficulty functioning in social life | 6 |
| Difficulty functioning at home / in family life | 7 |
| Lack of intimacy with partner **<PN: ALWAYS ANCHOR IN THIS POSITION>** | 8 |
| None of these | 9 |
| Unsure / don’t know | 10 |

1. What treatment(s) is this patient responding to?

**<PN: MULTI CODE> <PN: ASK ALL RESPONDENTS> <PN: SHOW ONLY LOCAL PRODUCT NAMES>**

| Molecule | Brands |  |
| --- | --- | --- |
| Agomelatine | <insert per specific market list> | 1 |
| Bupropion |  | 2 |
| Citalopram |  | 3 |
| Duloxetine |  | 4 |
| Escitalopram |  | 5 |
| Fluoxetine |  | 6 |
| Mirtazapine |  | 7 |
| Paroxetine |  | 8 |
| Sertraline |  | 9 |
| Venlafaxine |  | 10 |
| Vortioxetine |  | 11 |
| Atypical antidepressants |  | 12 |
| Benzodiazepines |  | 13 |
| Other drug therapy |  | 14 |
| Non-drug therapy (e.g. CBT) |  | 15 |
| No treatment or therapy prescribed |  | 16 |

1. Which of the following best describes the treatment decision made during this POST-ACUTE phase?

**<PN: MULTI CODE> <PN: ASK ALL RESPONDENTS WHO CODE 1-14 AT Q52>**

| The patient is newly diagnosed and was prescribed pharmacological treatment for the first time | 1 |
| --- | --- |
| A repeat prescription was given as the patient has been responding well to this treatment | 2 |
| The dosage/formulation of the treatment was changes to achieve a better response | 3 |
| Another antidepressant was added to their existing treatment to achieve a better response | 4 |
| A different medication was added to their existing treatment – such as an anti-psychotic or mood stabilizer to achieve a better response | 5 |
| The patient was switched from one pharmacological treatment to another | 6 |
| Pharmacological treatment was re-started after the patient went for a period without taking medication | 7 |

1. What are the symptoms that you most want to address with the treatment prescribed during this POST-ACUTE phase?

**<PN: MULTI CODE> <PN: ASK ALL RESPONDENTS – ONLY SHOW RESPONSES GIVEN AT Q49> <PN: ROTATE STATEMENT ORDER> <PN: ROTATE ORDER IN WHICH ‘GROUPS’ OF SYMPTOMS SHOWN AND ORDER OF INDIVIDUAL SYMPTOMS WITHIN GROUPS>**

| Feeling sad / Low | 1 |
| --- | --- |
| Lack of energy | 2 |
| Lack of interest in general | 3 |
| Unable to take pleasure in things | 4 |
| Low self esteem | 5 |
| Lacking in confidence | 6 |
| Feelings of guilt or worthlessness | 7 |
| Suicidal thoughts | 8 |
|  |  |
| Reduced activity | 9 |
| Difficulty sleeping | 10 |
| Feeling tired | 11 |
| Waking early in the morning | 12 |
| Feeling agitated / Restless | 13 |
| Weight loss | 14 |
| Loss of libido | 15 |
| Loss of appetite | 16 |
| Depression worst in the morning | 17 |
|  |  |
| Forgetfulness / difficulty remembering | 18 |
| Slowness of thinking | 19 |
| Slowness in physical movements | 20 |
| Difficulty concentrating | 21 |
| Difficulty to prioritise / make decisions | 22 |
| Difficulty in making plans | 23 |
|  |  |
| Other (please specify) **<PN: ANCHOR>** | 24 |
| No symptoms **<PN: ANCHOR>** | 25 |

1. Which ONE of the following best reflects your primary treatment goal during this POST-ACUTE phase?

**<PN: SINGLE CODE> <PN: ASK ALL RESPONDENTS> <PN: ROTATE STATEMENTS>**

| To elevate the patient’s mood | 1 |
| --- | --- |
| To reduce side effects | 2 |
| To return the patient to work | 3 |
| To return the patient to normal family life | 4 |
| To return the patient to normal social life | 5 |

1. Which of the following symptoms that this patient has experienced during this POST-ACUTE phase of depression, if any, can you NOT adequately treat?

**<PN: MULTI CODE> <PN: ROTATE STATEMENT ORDER - ONLY SHOW RESPONSES GIVEN AT Q49 > <PN: ROTATE ORDER IN WHICH ‘GROUPS’ OF SYMPTOMS SHOWN AND ORDER OF INDIVIDUAL SYMPTOMS WITHIN GROUPS>**

| Feeling sad / Low | 1 |
| --- | --- |
| Lack of energy | 2 |
| Lack of interest in general | 3 |
| Unable to take pleasure in things | 4 |
| Low self esteem | 5 |
| Lacking in confidence | 6 |
| Feelings of guilt or worthlessness | 7 |
| Suicidal thoughts | 8 |
|  |  |
| Reduced activity | 9 |
| Difficulty sleeping | 10 |
| Feeling tired | 11 |
| Waking early in the morning | 12 |
| Feeling agitated / Restless | 13 |
| Weight loss | 14 |
| Loss of libido | 15 |
| Loss of appetite | 16 |
| Depression worst in the morning | 17 |
|  |  |
| Forgetfulness / difficulty remembering | 18 |
| Slowness of thinking | 19 |
| Slowness in physical movements | 20 |
| Difficulty concentrating | 21 |
| Difficulty to prioritise / make decisions | 22 |
| Difficulty in making plans | 23 |
|  |  |
| Other (please specify) **<PN: ANCHOR>** | 24 |
| No symptoms – I can adequately treat them all **<PN: ANCHOR>** | 25 |

1. As far as you are aware, which of the following symptoms does the patient most want treated during this POST-ACUTE phase?

**<PN: MULTI CODE> <PN: ASK ALL RESPONDENT S- ONLY SHOW RESPONSES GIVEN AT Q49> <PN: ROTATE ORDER IN WHICH ‘GROUPS’ OF SYMPTOMS SHOWN AND ORDER OF INDIVIDUAL SYMPTOMS WITHIN GROUPS>**

| Feeling sad / Low | 1 |
| --- | --- |
| Lack of energy | 2 |
| Lack of interest in general | 3 |
| Unable to take pleasure in things | 4 |
| Low self esteem | 5 |
| Lacking in confidence | 6 |
| Feelings of guilt or worthlessness | 7 |
| Suicidal thoughts | 8 |
|  |  |
| Reduced activity | 9 |
| Difficulty sleeping | 10 |
| Feeling tired | 11 |
| Waking early in the morning | 12 |
| Feeling agitated / Restless | 13 |
| Weight loss | 14 |
| Loss of libido | 15 |
| Loss of appetite | 16 |
| Depression worst in the morning | 17 |
|  |  |
| Forgetfulness / difficulty remembering | 18 |
| Slowness of thinking | 19 |
| Slowness in physical movements | 20 |
| Difficulty concentrating | 21 |
| Difficulty to prioritise / make decisions | 22 |
| Difficulty in making plans | 23 |
|  |  |
| Other (please specify) **<PN: ANCHOR>** | 24 |
| None **<PN: ANCHOR>** | 25 |

1. In your opinion, to what extent is the patient experiencing difficulties with the following aspects during the post-acute phase:

**<PN: MUTLI CODE> <PN: ASK ALL RESPONDENTS> <PN: ROTATE STATEMENT GROUPS AND STATEMENTS WITHIN GROUPS>**

| No difficulty | Mild Difficulty | Moderate difficulty | Severe difficulty | Don’t know |
| --- | --- | --- | --- | --- |
| 1 | 2 | 3 | 4 | 5 |

| **AUTONOMY** |  |
| --- | --- |
| Taking responsibility for his/her household | 1 |
| Living on his/her own | 2 |
| Doing his/her shopping | 3 |
| Taking care of themselves (physical aspects, hygiene) | 4 |
| **OCCUPATIONAL FUNCTIONING** |  |
| Holding down a paid job | 5 |
| Accomplishing tasks as quickly as necessary | 6 |
| Working in the field in which he/she were educated | 7 |
| Maintaining the amount of money he/she earns in relation to their job position | 8 |
| Managing his/her expected work load | 9 |
| **COGNITIVE FUNCTIONING** |  |
| Ability to concentrate on a book, film | 10 |
| Ability to make mental calculations | 11 |
| Ability to solve a problem adequately | 12 |
| Ability to remember newly-learned names | 13 |
| Ability to learn new information | 14 |
| **FINANCIAL ISSUES** |  |
| Managing his/her own money | 15 |
| Spending his/her money in a balanced way | 16 |
| **INTERPERSONAL RELATIONSHIPS** |  |
| Maintaining a friendship or friendships | 17 |
| Participating in social activities | 18 |
| Having good relationships with people close to him/her | 19 |
| Living together with his/her family | 20 |
| Having satisfactory sexual relationships | 21 |
| Being able to defend his/her interests | 22 |
| **LEISURE TIME** |  |
| Doing exercise or participating in sport | 23 |
| Having hobbies or personal interests | 24 |

1. To what degree did you discuss each of the following issues with the patient during this POST-ACUTE phase?

**<PN: SINGLE CODE> <PN: ASK ALL RESPONDENTS>**

| Did not discuss | Discussed a little | Discussed a lot |
| --- | --- | --- |
| 1 | 2 | 3 |

| Treatment options | 1 |
| --- | --- |
| Impact of depression on family/home life | 2 |
| Impact of depression on social life (e.g. socialising with friends) | 3 |
| Impact of depression on work life | 4 |
| Side effects of existing treatments | 5 |
| Sid effects of new treatment options | 6 |

1. Which of the following topics, if any, did the patient raise with you at any point during this POST-ACUTE phase?

**<PN: MULTI CODE> <PN: ASK ALL RESPONDENTS>**

| Treatment options | 1 |
| --- | --- |
| Impact of depression on family/home life | 2 |
| Impact of depression on social life (e.g. socialising with friends) | 3 |
| Impact of depression on work life | 4 |
| Side effects of existing treatments | 5 |
| Sid effects of new treatment options | 6 |
| None of these | 7 |

1. How did the patient react to the decision to **<PN: INSERT TREATMENT DECISION FROM Q53>** that was made during this POST-ACUTE phase?

**<PN: SINGLE CODE> <PN: ASK ALL RESPONDENTS>**

| Very negatively |  |  |  |  |  | Very positively |
| --- | --- | --- | --- | --- | --- | --- |
| 1 | 2 | 3 | 4 | 5 | 6 | 7 |

1. Please indicate the extent to which the patient influenced your decision to **<PN: INSERT TREATMENT DECISION FROM Q53>**?

**<PN: SINGLE CODE> <PN: ASK ALL RESPONDENTS>**

| Not at all influential |  |  |  |  |  | Very influential |
| --- | --- | --- | --- | --- | --- | --- |
| 1 | 2 | 3 | 4 | 5 | 6 | 7 |

1. Please review each of the following statements about your management of the patient and indicate the degree to which you agree or disagree with each:

**<PN: SINGLE CODE> <PN: ASK ALL RESPONDENTS>**

| Disagree completely |  |  | Indifferent |  |  | Agree completely |
| --- | --- | --- | --- | --- | --- | --- |
| 1 | 2 | 3 | 4 | 5 | 6 | 7 |

| This patient is good at verbalising the symptoms they experience | 1 |
| --- | --- |
| I enjoy managing and treating this patient | 2 |
| This patient needs a treatment that will help them function better at work **<PN: DO NOT SHOW IF CODE 3-6 AT Q37>** | 3 |
| This patient needs a treatment that will help them function better in their social life (e.g. socialising with friends) | 4 |
| This patient needs a treatment that will help them function better at home / in family life | 5 |
| I find this patient extremely challenging to manage and treat | 6 |
| This patient suffers primarily from mood symptoms | 7 |
| Consultations with this patient are often difficult and unproductive | 8 |
| I wish this patient would play more of an active role in our consultations | 9 |
| It is just as important to treat this patient’s cognitive symptoms as it is their mood symptoms | 10 |
| This patient’s cognitive symptoms have not been controlled by medication quite as well as their mood symptoms have been controlled | 11 |
| This patient has never discussed cognitive symptoms with me | 12 |

**Section 5: Patient Case Record #3 ‘Remission’ (8 minutes)**

**<PN: PLEASE ROTATE THE ORDER IN WHICH SECTIONS 3,4 AND 5 ARE SHOWN TO THE RESPONDENT, SO THAT EACH DIFFERENT TYPE OF CASE RECORD IS ROTATED>**

**<PN: PLEASE PROGRAMME AT THE TOP OF EACH PAGE A NOTE TO REMIND THE RESPONDENT ABOUT WHICH CASE RECORD IS BEING DISCUSSED – ACUTE / POST-ACUTE / REMISSION>**

To begin with we would like you to focus on **<PN: ROTATE PCR 1/2/3 ORDER>**

Next we would like you to focus on **<PN: ROTATE PCR 1/2/3 ORDER>**

**<PN: DISPLAY DEFINITION>**

…an MDD **<PN: Mexico MDD = ‘Depression’/ US and Can ‘MDD’, EU ‘MDE’>** patient who is in the **REMISSION** phase of the condition. By **remission phase** we mean: the patient feels better and experiences a significant reduction in symptoms compared to the acute or post-acute phase. Some residual symptoms may persist but are significantly fewer in number and severity compared to other phases

**Background information about the patient**

1. How long ago did this consultation with the patient take place *(please enter as appropriate)*?

**<PN: SINGLE CODE> <PN: ASK ALL RESPONDENTS>**

|  |  | Weeks |  |  | Months |  |  | Years |
| --- | --- | --- | --- | --- | --- | --- | --- | --- |

1. How old is this patient?

**<PN: NUMERIC. RANGE 18-90 / 25-70 in the US> <PN: ASK ALL RESPONDENTS>**

|  |  |
| --- | --- |

1. Indicate the patient’s gender

**<PN: SINGLE CODE> <PN: ASK ALL RESPONDENTS>**

| Male | 1 |
| --- | --- |
| Female | 2 |

1. Which of the following best describes the patient’s highest level of education? <**PN: SINGLE CODE> <PN: ASK ALL RESPONDENTS>**

| Post-graduate | 1 |
| --- | --- |
| Undergraduate | 2 |
| High School Graduate | 3 |
| Vocational Qualification | 4 |
| No Qualifications | 5 |
| Other | 6 |
| Don’t know | 7 |

1. Which of the following best describes the patient’s current working status? **<PN: SINGLE CODE> <PN: ASK ALL RESPONDENTS>**

| Full-time (30 or more hours per week) | 1 | **Go to Q69** |
| --- | --- | --- |
| Part-time | 2 | **Go to Q69** |
| Retired | 3 | **Go to Q70** |
| Homemaker/ Stay-at-home parent | 4 | **Go to Q70** |
| Student | 5 | **Go to Q70** |
| Unemployed | 6 | **Go to Q70** |

1. Which of the following occupational groups best reflects the type of job the patient does

**<PN: SINGLE CODE> <PN: ASK ALL RESPONDENTS>**

| **Semi or unskilled manual work** (e.g. Manual workers, apprentices to be skilled trades, Shop assistants etc.) | 1 |
| --- | --- |
| **Skilled manual worker** (e.g. Plumber, Lorry driver, Mechanic etc.) | 2 |
| **Supervisory or clerical** junior managerial/administrative (e.g. Office worker, Student Doctor, Foreman etc.) | 3 |
| **Intermediate managerial/ professional/ administrative** (e.g. Newly qualified doctor, Solicitor, Middle manager in large organisation) | 4 |
| **Higher managerial/ professional/ administrative** (e.g. Established doctor, Solicitor, Board Director in a large organisation, top level civil servant) | 5 |
| **Student** | 6 |
| **Casual worker** (not in permanent employment) | 7 |
| **Homemaker/ stay at home parent** | 8 |
| **Retired** | 9 |
| **Unemployed or not working due to long-term sickness** | 10 |
| **Other** | 11 |

1. Approximately how long ago was this patient diagnosed with MDD **<PN: Mexico MDD = ‘Depression’/ US and Can ‘MDD’, EU ‘MDE’>**  *(please enter as appropriate)*?

**<PN: NUMERIC. RANGE 0-90> <PN: ASK ALL RESPONDENTS>**

|  |  | Years |  |  |  | Months |  |  | Don’t know |
| --- | --- | --- | --- | --- | --- | --- | --- | --- | --- |

1. For approximately how long has this patient been in this **remission** phase of MDD **<PN: Mexico MDD = ‘Depression’/ US and Can ‘MDD’, EU ‘MDE’>** *(please enter as appropriate)*?

**<PN: NUMERIC> <PN: ASK ALL RESPONDENTS>**

|  |  | Weeks |  |  |  | Months |  |  | Years |
| --- | --- | --- | --- | --- | --- | --- | --- | --- | --- |

1. Did you personally initiate the treatment that this patient is currently using whilst in REMISSION?

**<PN: SINGLE CODE> <PN: ASK ALL RESPONDENTS>**

| Yes I did | 1 |
| --- | --- |
| No I did not, but the patient is taking a treatment | 2 |
| No – because the patient is not taking a treatment | 3 |

1. What type of doctor initiated the treatment the patient is taking whilst in REMISSION?

**<PN: SINGLE CODE> <PN: ASK ALL RESPONDENTS CODING 2 AT Q72>**

| Primary care physician | 1 |
| --- | --- |
| Psychiatrist | 2 |
| Neurologist | 3 |
| Geriatrician | 4 |
| Internal Medicine | 5 |
| Another specialist | 6 |
| Other | 7 |
| Don’t know | 8 |

1. Did this patient present directly to you or were they referred to you from another physician?

**<PN: SINGLE CODE> <PN: ASK ALL RESPONDENTS>**

| Presented directly to me | 1 |
| --- | --- |
| Were referred by another physician | 2 |

1. What type of physician referred the patient on to you?

**<PN: SINGLE CODE> <PN: ASK ALL RESPONDENTS WHO CODE 2 AT Q74>**

| Primary care physician | 1 |
| --- | --- |
| Psychiatrist | 2 |
| Neurologist | 3 |
| Psychologist | 4 |
| Another specialist | 5 |
| Other | 6 |
| Don’t know | 7 |

1. For approximately how long have you been managing this patient?

**<PN: SINGLE CODE> <PN: ASK ALL RESPONDENTS>**

|  |  | Weeks |  |  | Months |  |  | Years |
| --- | --- | --- | --- | --- | --- | --- | --- | --- |

1. Is this the first REMISSION phase of MDD **<PN: Mexico MDD = ‘Depression’/ US and Can ‘MDD’, EU ‘MDE’>** that the patient has had or have they had previous remission phases?

**<PN: SINGLE CODE> <PN: ASK ALL RESPONDENTS>**

| First time | 1 | Go to Q79 |
| --- | --- | --- |
| Previous REMISSION phases | 2 | Go to Q78 |

1. Approximately how many previous REMISSION phases (excluding the current remission phase) have they had prior to the most recent REMISSION phase?

**<PN: SINGLE CODE> <PN: ASK ALL RESPONDENTS>**

|  |  |  | REMISSION phases |
| --- | --- | --- | --- |

| Don’t know | 1 |
| --- | --- |

1. Has the patient’s working status changed because of this REMISSION phase of depression?

<**PN: MUTLI CODE> <PN: ASK ALL RESPONDENTS>**

| **Yes** – they have had to **reduce their working hours** | 1 |
| --- | --- |
| **Yes** – they have had to **take a** **less demanding job** | 2 |
| **Yes** – They have had to **take sick leave** | 3 |
| **Yes** – They have had to **stop working** | 4 |
| **No** – their working status **has not changed** | 5 |
| Don’t know | 6 |

**Information about REMISSION phase**

Please remember that with this patient we are focusing on the **REMISSION** phase of the condition. By **remission phase** we mean: the patient feels better and experiences a significant reduction in symptoms compared to the acute or post-acute phase. Some residual symptoms may persist but are significantly fewer in number and severity compared to other phases

1. Which residual symptoms did this patient experience during this **REMISSION** phase?

**<PN: MULTI CODE> <PN: ASK ALL RESPONDENTS> <PN: ROTATE ORDER IN WHICH ‘GROUPS’ OF SYMPTOMS SHOWN AND ORDER OF INDIVIDUAL SYMPTOMS WITHIN GROUPS>**

| Feeling sad / Low | 1 |
| --- | --- |
| Lack of energy | 2 |
| Lack of interest in general | 3 |
| Unable to take pleasure in things | 4 |
| Low self esteem | 5 |
| Lacking in confidence | 6 |
| Feelings of guilt or worthlessness | 7 |
| Suicidal thoughts | 8 |
|  |  |
| Reduced activity | 9 |
| Difficulty sleeping | 10 |
| Feeling tired | 11 |
| Waking early in the morning | 12 |
| Feeling agitated / Restless | 13 |
| Weight loss | 14 |
| Loss of libido | 15 |
| Loss of appetite | 16 |
| Depression worst in the morning | 17 |
|  |  |
| Forgetfulness / difficulty remembering | 18 |
| Slowness of thinking | 19 |
| Slowness in physical movements | 20 |
| Difficulty concentrating | 21 |
| Difficulty to prioritise / make decisions | 22 |
| Difficulty in making plans | 23 |
|  |  |
| Other (please specify) **<PN: ANCHOR>** | 24 |
| No symptoms **<PN: ANCHOR>** | 25 |

1. Overall, how severe would you rate the residual symptoms that the patient is experiencing in this REMISSION phase?

**<PN: SINGLE CODE> <PN: ASK ALL RESPONDENTS WHO CODE 1-24 at Q80>**

| Not at all severe |  |  |  |  |  | Extremely severe |
| --- | --- | --- | --- | --- | --- | --- |
| 1 | 2 | 3 | 4 | 5 | 6 | 7 |

1. Which of the following has the patient experienced during this REMISSION phase of depression?

**<PN: MULTI CODE> <PN: ASK ALL RESPONDENTS>**

| Withdrawal from social life | 1 |
| --- | --- |
| Relationship breakdown | 2 |
| Loss of employment | 3 |
| Inability to find employment | 4 |
| Difficulty functioning effectively at work or in studies | 5 |
| Difficulty functioning in social life | 6 |
| Difficulty functioning at home / in family life | 7 |
| Lack of intimacy with partner **<PN: ALWAYS ANCHOR IN THIS POSITION>** | 8 |
| None of these | 9 |
| Unsure / don’t know | 10 |

1. What treatment(s) did you prescribe to the patient during this **REMISSION** phase?

**<PN: MULTI CODE> <PN: ASK ALL RESPONDENTS> <PN: SHOW ONLY LOCAL PRODUCT NAMES>**

| Molecule | Brands |  |
| --- | --- | --- |
| Agomelatine | <insert per specific market list> | 1 |
| Bupropion |  | 2 |
| Citalopram |  | 3 |
| Duloxetine |  | 4 |
| Escitalopram |  | 5 |
| Fluoxetine |  | 6 |
| Mirtazapine |  | 7 |
| Paroxetine |  | 8 |
| Sertraline |  | 9 |
| Venlafaxine |  | 10 |
| Vortioxetine |  | 11 |
| Atypical antidepressants |  | 12 |
| Benzodiazepines |  | 13 |
| Other drug therapy |  | 14 |
| Non-drug therapy (e.g. CBT) |  | 15 |
| No treatment or therapy prescribed |  | 16 |

1. Which of the following best describes the treatment decision made during this REMISSION phase **<PN: INSERT PRODUCT(S) SELECTED FROM Q83>**?

**<PN: MULTI CODE> <PN: ASK ALL RESPONDENTS WHO CODE 1-14 AT Q83>**

| The patient is newly diagnosed and was prescribed pharmacological treatment for the first time | 1 |
| --- | --- |
| A repeat prescription was given as the patient has been responding well to this treatment | 2 |
| The dosage/formulation of the treatment was changes to achieve a better response | 3 |
| Another antidepressant was added to their existing treatment to achieve a better response | 4 |
| A different medication was added to their existing treatment – such as an anti-psychotic or mood stabilizer to achieve a better response | 5 |
| The patient was switched from one pharmacological treatment to another | 6 |
| Pharmacological treatment was re-started after the patient went for a period without taking medication | 7 |

1. What are the residual symptoms that you most want to address with the treatment prescribed during this REMISSION phase?

**<PN: MULTI CODE> <PN: ASK ALL RESPONDENTS EXCLUDING THOSE WHO CODE 24 AT Q80 – ONLY SHOW RESPONSES GIVEN AT Q80> <PN: ROTATE STATEMENT ORDER> <PN: ROTATE ORDER IN WHICH ‘GROUPS’ OF SYMPTOMS SHOWN AND ORDER OF INDIVIDUAL SYMPTOMS WITHIN GROUPS>**

| Feeling sad / Low | 1 |
| --- | --- |
| Lack of energy | 2 |
| Lack of interest in general | 3 |
| Unable to take pleasure in things | 4 |
| Low self esteem | 5 |
| Lacking in confidence | 6 |
| Feelings of guilt or worthlessness | 7 |
| Suicidal thoughts | 8 |
|  |  |
| Reduced activity | 9 |
| Difficulty sleeping | 10 |
| Feeling tired | 11 |
| Waking early in the morning | 12 |
| Feeling agitated / Restless | 13 |
| Weight loss | 14 |
| Loss of libido | 15 |
| Loss of appetite | 16 |
| Depression worst in the morning | 17 |
|  |  |
| Forgetfulness / difficulty remembering | 18 |
| Slowness of thinking | 19 |
| Slowness in physical movements | 20 |
| Difficulty concentrating | 21 |
| Difficulty to prioritise / make decisions | 22 |
| Difficulty in making plans | 23 |
|  |  |
| Other (please specify) **<PN: ANCHOR>** | 24 |
| No symptoms **<PN: ANCHOR>** | 25 |

1. Which ONE of the following best reflects your primary treatment goal during this REMISSION phase?

**<PN: SINGLE CODE> <PN: ASK ALL RESPONDENTS> <PN: ROTATE STATEMENTS>**

| To elevate the patient’s mood | 1 |
| --- | --- |
| To reduce side effects | 2 |
| To return the patient to work | 3 |
| To return the patient to normal family life | 4 |
| To return the patient to normal social life | 5 |

1. Which of the following residual symptoms that this patient has experienced during this REMISSION phase of depression, if any, can you NOT adequately treat?

**<PN: MULTI CODE> <PN: ROTATE STATEMENT ORDER - ONLY SHOW RESPONSES GIVEN AT Q80> <PN: ROTATE ORDER IN WHICH ‘GROUPS’ OF SYMPTOMS SHOWN AND ORDER OF INDIVIDUAL SYMPTOMS WITHIN GROUPS>**

| Feeling sad / Low | 1 |
| --- | --- |
| Lack of energy | 2 |
| Lack of interest in general | 3 |
| Unable to take pleasure in things | 4 |
| Low self esteem | 5 |
| Lacking in confidence | 6 |
| Feelings of guilt or worthlessness | 7 |
| Suicidal thoughts | 8 |
|  |  |
| Reduced activity | 9 |
| Difficulty sleeping | 10 |
| Feeling tired | 11 |
| Waking early in the morning | 12 |
| Feeling agitated / Restless | 13 |
| Weight loss | 14 |
| Loss of libido | 15 |
| Loss of appetite | 16 |
| Depression worst in the morning | 17 |
|  |  |
| Forgetfulness / difficulty remembering | 18 |
| Slowness of thinking | 19 |
| Slowness in physical movements | 20 |
| Difficulty concentrating | 21 |
| Difficulty to prioritise / make decisions | 22 |
| Difficulty in making plans | 23 |
|  |  |
| Other (please specify) **<PN: ANCHOR>** | 24 |
| No residual symptoms – I can adequately treat them all **<PN: ANCHOR>** | 25 |

1. As far as you are aware, which of the following residual symptoms does this patient most want treated during this REMISSION phase?

**<PN: MULTI CODE> <PN: ASK ALL RESPONDENTS - ONLY SHOW RESPONSES GIVEN AT Q80> <PN: ROTATE ORDER IN WHICH ‘GROUPS’ OF SYMPTOMS SHOWN AND ORDER OF INDIVIDUAL SYMPTOMS WITHIN GROUPS>**

| Feeling sad / Low | 1 |
| --- | --- |
| Lack of energy | 2 |
| Lack of interest in general | 3 |
| Unable to take pleasure in things | 4 |
| Low self esteem | 5 |
| Lacking in confidence | 6 |
| Feelings of guilt or worthlessness | 7 |
| Suicidal thoughts | 8 |
|  |  |
| Reduced activity | 9 |
| Difficulty sleeping | 10 |
| Feeling tired | 11 |
| Waking early in the morning | 12 |
| Feeling agitated / Restless | 13 |
| Weight loss | 14 |
| Loss of libido | 15 |
| Loss of appetite | 16 |
| Depression worst in the morning | 17 |
|  |  |
| Forgetfulness / difficulty remembering | 18 |
| Slowness of thinking | 19 |
| Slowness in physical movements | 20 |
| Difficulty concentrating | 21 |
| Difficulty to prioritise / make decisions | 22 |
| Difficulty in making plans | 23 |
|  |  |
| Other (please specify) **<PN: ANCHOR>** | 24 |
| None **<PN: ANCHOR>** | 25 |

1. In your opinion, to what extent is the patient experiencing difficulties with the following aspects during the remission phase:

**<PN: MUTLI CODE> <PN: ASK ALL RESPONDENTS> <PN: ROTATE STATEMENT GROUPS AND STATEMENTS WITHIN GROUPS>**

| No difficulty | Mild Difficulty | Moderate difficulty | Severe difficulty | Don’t know |
| --- | --- | --- | --- | --- |
| 1 | 2 | 3 | 4 | 5 |

| **AUTONOMY** |  |
| --- | --- |
| Taking responsibility for his/her household | 1 |
| Living on his/her own | 2 |
| Doing his/her shopping | 3 |
| Taking care of themselves (physical aspects, hygiene) | 4 |
| **OCCUPATIONAL FUNCTIONING** |  |
| Holding down a paid job | 5 |
| Accomplishing tasks as quickly as necessary | 6 |
| Working in the field in which he/she were educated | 7 |
| Maintaining the amount of money he/she earns in relation to their job position | 8 |
| Managing his/her expected work load | 9 |
| **COGNITIVE FUNCTIONING** |  |
| Ability to concentrate on a book, film | 10 |
| Ability to make mental calculations | 11 |
| Ability to solve a problem adequately | 12 |
| Ability to remember newly-learned names | 13 |
| Ability to learn new information | 14 |
| **FINANCIAL ISSUES** |  |
| Managing his/her own money | 15 |
| Spending his/her money in a balanced way | 16 |
| **INTERPERSONAL RELATIONSHIPS** |  |
| Maintaining a friendship or friendships | 17 |
| Participating in social activities | 18 |
| Having good relationships with people close to him/her | 19 |
| Living together with his/her family | 20 |
| Having satisfactory sexual relationships | 21 |
| Being able to defend his/her interests | 22 |
| **LEISURE TIME** |  |
| Doing exercise or participating in sport | 23 |
| Having hobbies or personal interests | 24 |

1. To what degree did you discuss each of the following issues with the patient at the during this REMISSION phase?

**<PN: SINGLE CODE> <PN: ASK ALL RESPONDENTS>**

| Did not discuss | Discussed a little | Discussed a lot |
| --- | --- | --- |
| 1 | 2 | 3 |

| Treatment options | 1 |
| --- | --- |
| Impact of depression on family/home life | 2 |
| Impact of depression on social life (e.g. socialising with friends) | 3 |
| Impact of depression on work life | 4 |
| Side effects of existing treatments | 5 |
| Sid effects of new treatment options | 6 |

1. Which of the following topics, if any, did the patient raise with you at any point during this REMISSION phase?

**<PN: MULTI CODE> <PN: ASK ALL RESPONDENTS>**

| Treatment options | 1 |
| --- | --- |
| Impact of depression on family/home life | 2 |
| Impact of depression on social life (e.g. socialising with friends) | 3 |
| Impact of depression on work life | 4 |
| Side effects of existing treatments | 5 |
| Sid effects of new treatment options | 6 |
| None of these | 7 |

1. How did the patient react to the decision to **<PN: INSERT TREATMENT DECISION FROM Q84>** that was made during this REMISSION phase?

**<PN: SINGLE CODE> <PN: ASK ALL RESPONDENTS>**

| Very negatively |  |  |  |  |  | Very positively |
| --- | --- | --- | --- | --- | --- | --- |
| 1 | 2 | 3 | 4 | 5 | 6 | 7 |

1. Please indicate the extent to which the patient influenced your decision to **<PN: INSERT TREATMENT DECISION FROM Q84>**?

**<PN: SINGLE CODE> <PN: ASK ALL RESPONDENTS>**

| Not at all influential |  |  |  |  |  | Very influential |
| --- | --- | --- | --- | --- | --- | --- |
| 1 | 2 | 3 | 4 | 5 | 6 | 7 |

1. Please review each of the following statements about your management of the patient and indicate the degree to which you agree or disagree with each:

**<PN: SINGLE CODE> <PN: ASK ALL RESPONDENTS>**

| Disagree completely |  |  | Indifferent |  |  | Agree completely |
| --- | --- | --- | --- | --- | --- | --- |
| 1 | 2 | 3 | 4 | 5 | 6 | 7 |

| This patient is good at verbalising the symptoms they experience | 1 |
| --- | --- |
| I enjoy managing and treating this patient | 2 |
| This patient needs a treatment that will help them function better at work **<PN: DO NOT SHOW IF CODE 3-6 AT Q68>** | 3 |
| This patient needs a treatment that will help them function better in their social life (e.g. socialising with friends) | 4 |
| This patient needs a treatment that will help them function better at home / in family life | 5 |
| I find this patient extremely challenging to manage and treat | 6 |
| This patient suffers primarily from mood symptoms | 7 |
| Consultations with this patient are often difficult and unproductive | 8 |
| I wish this patient would play more of an active role in our consultations | 9 |
| It is just as important to treat this patient’s cognitive symptoms as it is their mood symptoms | 10 |
| This patient’s cognitive symptoms have not been controlled by medication quite as well as their mood symptoms have been controlled | 11 |
| This patient has never discussed cognitive symptoms with me | 12 |

**Section 6: Attitudes to depression and treatment (1 minute)**

1. Which one of the following statements best reflects your prescribing of new drugs:

**<PN: SINGLE CODE> <PN: ASK ALL RESPONDENTS>**

| I usually prescribe a new drug only after it has become standard therapy | 1 |
| --- | --- |
| I usually prescribe a new drug only after I have understood my colleagues’ experiences with it | 2 |
| I usually prescribe a new drug quite soon after it has been approved | 3 |
| I am usually one of the first physicians to prescribe a new drug | 4 |

**Thank and close survey**

**Product References (for programming):**

| Molecule | Brazil | Canada | Mexico | S.K | USA | France | Italy | Spain |
| --- | --- | --- | --- | --- | --- | --- | --- | --- |
| Agomelatine | <to be inserted> | - |  | Not available |  | Valdoxan | Thymanax; Valdoxan | Thymanax; Valdoxan |
| Bupropion |  | Budeprion; Bupropion; Wellbutrin |  |  |  | - | Elontril; Wellbutrin | Elontril |
| Citalopram |  | Celexa; Cipramil; Citalopram; Ran; Ran-Citalo; Talohexal |  | Not available |  | Citalopram; Seropram | Citalopram; Citisint; Elopram; Felipram; Feliximir; Frimaind; Kaidor; Lampopram; Marpram; Percitale; Pramexyl; Return; Ricap; Seropram; Sintopram; Verisan; Vivipram | Calton; Citaolpram; Citalvir; Genprol; Prisdal; Relapaz; Seregra; Seropram |
| Duloxetine |  | Cymbalta |  |  |  | Cymbalta | Cymbalta; Xeristar | Cymbalta; Xeristar |
| Escitalopram |  | Cipralex |  |  |  | Seroplex | Cipralex; Entact | Cipralex; Escilan; Escimylan; Escitalopram; Esertia; Heipram |
| Fluoxetine |  | Auscap; Erocap; Fluohexal; Fluoxetine; FXT 20; FXT 40; Lovan; Prozac; Zactin |  |  |  | Fluoxetine; Prozac | Azur; Clexiclor; Cloriflox; Deprexen; Diesan; Flotina; Fluoxetine; Prozac; Xeredien | Adofen; Astrin; Fluoxetine; Lecimar; Luramon; Nodepe; Prozac; Reneuron |
| Mirtazapine |  | Avanza; Mirtazapine; Remeron |  |  |  | Mirtazapine; Norset | Mirtazapine; Remeron; Zatimar | Afloyan; Mirtamylan; Mirtazapine; Rexer; Vastat |
| Paroxetine |  | Aropax; Paroxetine; Paxil; Paxtine |  |  |  | Deroxat; Divarius;Paroxatine | Dapagut; Daparox; Dropaxin; Eutimil; Paroxetine; Serestill; Serupin; Seroxat; Stiliden | Arapaxel; Casbol; Daparox;; Frosinor; Motivan; Paroxteine; Seroxat; Xetin; Zuria |
| Sertraline |  | Sertraline; Zoloft |  |  |  | Sertraline; Zoloft | Serad; Sertraline; Tatig; Tralforin; Tralisen; Zoloft | Altisben; Aremis; Aserin; Besitran; Sertraline |
| Desvenlafaxine |  |  |  |  |  |  |  | Pristiq |
| Venlafaxine |  | Effexor; Venlafaxine; Venlafaxine SR |  |  |  | Efexor; Venlafaxine | Efexor; Faxine; Venlafaxine | Arafaxina; Conervin; Dislaven; Dobupal; Flaxen; Levest; Vandral; Venlabrain; Venlafaxine; Venlamylan; Zarelis |
| Vortioxetine | Brintellix | Trintellix | Brintellix | Brintellix | Trintellix | Brintellix | Brintellix | Brintellix |

**Quotas:**

| Country | Quota | |
| --- | --- | --- |
|  | **Psychiatrists** | **Primary care Practitioners** |
| **Brazil** | **75** | **50** |
| **Canada** | **75** | **50** |
| **Mexico** | **75** | **50** |
| **South Korea** | **125** | **-** |
| **USA** | **100** | **50** |
| **France** | **75** | **50** |
| **Italy** | **75** | **50** |
| **Spain** | **75** | **50** |
