# Supplementary material for: Differences in Perceptions of Major Depressive Disorder Symptoms and Treatment Priorities Between Patients and Health Care Providers Across the Acute, Post-Acute, and Remission Phases of Depression
Source: Front Psychiatry. 2019 May 21;10:335. doi: 10.3389/fpsyt.2019.00335 (PMC6537882; doi:10.3389/fpsyt.2019.00335)
Supplement: Supplementary file 2 [file DataSheet_3.docx]

**Supplementary figures appendix D**

**Supplementary Figure 1. Mean FAST score for each domain by patient cohort**

Mean FAST score

Q22 (Patient) To what extent are you experiencing difficulties with the following aspects during this phase of depression? Baseline: All patients excluding those who stated ‘Don’t know’ to 5 or more statements. Acute n=406, Post-acute n=767, Remission n=773.

FAST, Functional Assessment Short Test; HCP, health care professional; n, subset of population.

**Supplementary Figure 2. Mean FAST score for each domain by HCP-assessed cohort**


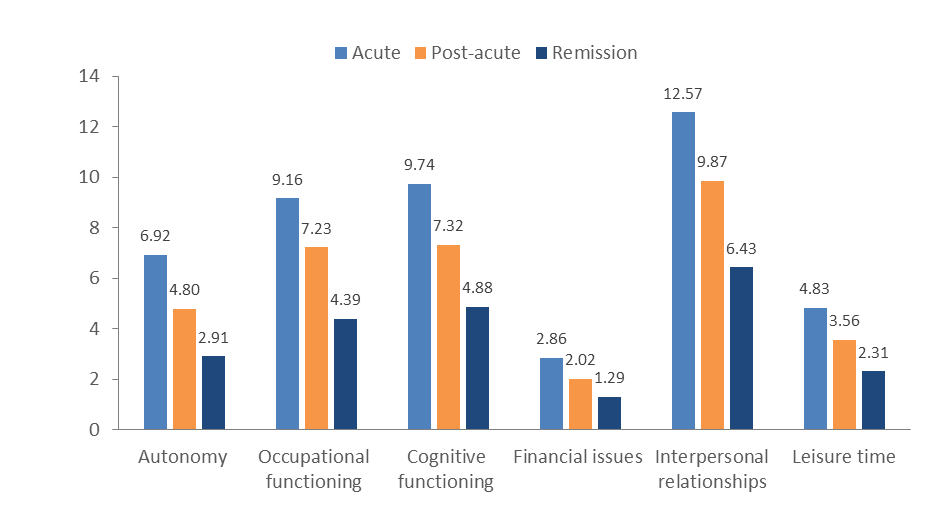


Mean FAST score

Q27/58/89 (HCP) To what extent is the patient experiencing difficulties with the following aspects during this phase of depression?

Baseline: All HCP patient case records excluding those who stated ‘Don’t know’ to 5 or more statements. Acute n=1005, Post-acute n=1017, Remission n=1020.

FAST, Functional Assessment Short Test; HCP, health care professional; n, subset of population.
